# Confirmation bias leads to overestimation of losses of woody plant foliage to insect herbivores in tropical regions

**DOI:** 10.7717/peerj.709

**Published:** 2014-12-23

**Authors:** Mikhail V. Kozlov, Vitali Zverev, Elena L. Zvereva

**Affiliations:** Section of Ecology, Department of Biology, University of Turku, Turku, Finland

**Keywords:** Brazil, Background herbivory, Miners, Defoliating insects, Consumed leaf area, Plant-herbivore interactions, Research bias, Gallers

## Abstract

Confirmation bias, i.e., the tendency of humans to seek out evidence in a manner that confirms their hypotheses, is almost overlooked in ecological studies. For decades, insect herbivory was commonly accepted to be highest in tropical regions. By comparing the data collected blindly (when the observer was not aware of the research hypothesis being tested) with the results of non-blind studies (when the observer knew what results could be expected), we tested the hypothesis that the records made in the tropics could have overestimated community-wide losses of plant foliage to insects due to the confirmation bias. The average loss of leaf area of woody plants to defoliating insects in Brazil, when measured by a blind method (1.11%), was significantly lower than the loss measured in non-blind studies, both original (5.14%) and published (6.37%). We attribute the overestimation of the community-wide losses of plant foliage to insects in non-blind studies to the unconsciously preconceived selection of study species with higher-than-average levels of herbivory. Based on our findings, we urge for caution in obtaining community-wide characteristics from the results of multiple single-species studies. Our data suggest that we may need to revise the paradigm of the highest level of background insect herbivory in the tropical regions. More generally, we argue that more attention should be paid by ecologists to the problem of biases occurring at the pre-publication phases of the scientific research and, consequently, to the development and the wide application of methods that avoid biases occurring due to unconscious psychological processes.

## Introduction

The relationships between plants and herbivores are among the most intensively studied biotic interactions (Tylianakis et al., 2008; Jamieson et al., 2012), and the amount of plant biomass consumed by herbivores is the key characteristic of the intensity of these interactions. It is commonly accepted that herbivores consume, on average, 18 percent of the biomass produced annually in terrestrial ecosystems (Cyr & Pace, 1993), and this value is widely cited as the proof of the substantial impact of herbivores on plants (e.g., Polis, 1999).

At the global scale, the larger part of herbivory is attributed to insects—‘the little things that run the World’ (Wilson, 1987). Explicitly or implicitly, the numerical values reflecting the pressure which herbivorous insects impose on plants are among the cornerstones of numerous hypotheses/theories related to insect-plant relationships, such as the ‘green world’ hypothesis (Hairston, Smith & Slobodkin, 1960; Polis, 1999) the exploitation ecosystem hypothesis (Oksanen et al., 1981; Polis, 1999), the Janzen–Connell hypothesis (Janzen, 1970), the optimal defence theory (Rhoades, 1979), the growth–differentiation balance hypothesis (Herms & Mattson, 1992) and many others, as well as of theories explaining evolution of plant traits (Coley & Aide, 1991) and formation of biogeographical patterns (Moles et al., 2011).

The data on losses of woody plant foliage to defoliating insects, summarized in several review papers (Springett, 1978; Whittaker & Warrington, 1985; Landsberg & Ohmart, 1989; Coley & Aide, 1991; Coley & Barone, 1996; Schowalter & Lowman, 1999; Dirzo & Boege, 2008), have been collected from a limited number of plant species during different years, and the question arises whether these data are representative of entire plant communities. However, the pool of primary data regarding plant losses to insects that served as the basis for numerous generalizations has never been subjected to rigorous examination to see whether these data provide unbiased community-wide estimates of the levels of background herbivory, i.e., of the amounts of plant biomass consumed by insects when their populations are at their ‘normal’ (non-outbreak) densities. The diversity in methodologies of data collection was recently suggested as one possible reason behind the lack of consistency among studies exploring the levels of herbivory along environmental gradients (Andrew, Roberts & Hill, 2012).

A number of biases occurring in the planning, data collection, analysis and publication phases of the scientific research are known to substantially influence their outcomes (Pannucci & Wilkins, 2010). Confirmation, observer or expectancy bias—the tendency of humans to seek out evidence and interpret it in a manner that confirms their existing ideas and hypotheses (Rosenthal, 1976; Nickerson, 1998)—is a well-documented phenomenon in psychology and cognitive science. This bias results primarily from automatic processes occurring unintentionally (Hergovich, Schott & Burger, 2010). Within biological disciplines, confirmation bias has received sufficient attention only in studies of animal behaviour (Marsh & Hanlon, 2007; Von Wilgenburg & Elgar, 2013). In ecological studies, the bias introduced by researchers was termed ‘research bias’ and was defined as the tendency to collect data on organisms or under conditions in which one has a reasonable expectation of detecting significant effects (Gurevitch & Hedges, 1999). In contrast to publication bias, where the influence on the understanding of ecological processes is widely appreciated (Jennions et al., 2013), the occurrence and importance of biases introduced at pre-publication stages of ecological research have received little attention (but see Kozlov & Zvereva, 2009; Koricheva, Jennions & Lau, 2013). This is especially true for confirmation bias, which occurs due to unconscious psychological processes (Hergovich, Schott & Burger, 2010) and thus differs from the bias driven by ‘reasonable expectations’ of the researcher (Gurevitch & Hedges, 1999).

Confirmation bias can be revealed by comparing the results of studies conducted blind (i.e., when the observer was not aware of the research hypothesis being tested) with the results of non-blind studies (i.e., when the observer knew what results could be expected) (Von Wilgenburg & Elgar, 2013). To our knowledge, no such comparisons have been performed to date in ecological research. We therefore established the present study to compare field estimates of losses of plant foliage to insects obtained by blind and non-blind methods in order to test whether confirmation bias occurs in studies of insect-plant relationships, and whether it affects our knowledge regarding the proportion of plant foliage consumed by herbivorous insects in the tropics.

The selection of plant species and study sites for measurements of foliar losses to herbivores, as well as of the time of the measurements, may have been affected by the researcher’s expectations or preconceptions, depending on both the researcher’s personal experience and on the hypothesis/theory which this researcher believed to be true. In particular, insect herbivory was commonly accepted for decades to be highest in tropical regions (Coley & Aide, 1991; Coley & Barone, 1996; Schowalter & Lowman, 1999), and this idea formed the foundation for a number of ecological hypotheses and generalizations, such as latitudinal changes in strength of biotic interactions (reviewed by Schemske et al., 2009) and geographic variation in the evolution of plant defences (Coley & Aide, 1991; Coley & Barone, 1996; Moles et al., 2011). Therefore, we suggest that records made in the tropics could have overestimated the community-wide losses of plant foliage to insects due to confirmation bias, i.e., due to unconscious selection for plant species showing higher-than-average herbivore damage to confirm the influential theory. Based on this hypothesis, we predicted that (1) the average foliar losses of woody plants calculated from published studies or measured in plant species that have been non-blindly selected by one of the authors of the present study will be higher than the estimate obtained from blindly selected plant species; and (2) within the data collected non-blindly, the average foliar losses of woody plants calculated from published studies (that generally have been conducted by local researches) will be higher than those measured by one of the authors of the present study who has no *a priori* knowledge of either plants or herbivores of the study area. In particular, the local researchers may know which localities and tree species are more likely to show high herbivore damage and design their studies accordingly. In addition to testing these predictions, we checked whether the published values of foliar losses of woody plants to insects may have been affected by reporting or publication bias.

## Material and Methods

### General approach

We compared the point data (i.e., measured at a single point in time) on damage of woody plant foliage by insects that were collected in five different ways (Table 1). The first data set (DS1 hereafter) was compiled from publications reporting this damage on the basis of field studies conducted in Brazil (Appendix S1). We presumed that researchers who conducted these studies possessed extensive knowledge on both plants and insects of the study region, and therefore their selection of study objects, study sites and timing of measurements may have been affected by both this knowledge and by their expectations based on the widespread opinion that plant losses to herbivores are high in the tropics. The second data set (DS2 hereafter) was collected in Brazil by one of the authors (MVK) from several abundant plant species (Appendix S2). The third data set (DS3 hereafter) consists of photographs taken for identification of plant species, foliar damage of which was assessed by MVK (DS2). Plant losses estimated from these images (DS3) were compared with the results of field measurements (DS2) to check the reliability of our method of image analysis. These three data sets (DS1–DS3) were all based on non-blind data collection. However, we presumed that the selection of study species by local scientists was more influenced by prejudice than was the selection of study species by MVK due to his limited knowledge of local flora and fauna. Additionally, selection of study sites and time of sampling by MVK was based on factors not related to the topic of the present study. The fourth data set (DS4 hereafter) consisted of nature, wildlife and portrait photographs taken by VZ and ELZ with a Canon 60D camera for non-scientific purposes (i.e., holiday snaps) in the same habitats, where MVK conducted the field measurements of insect folivory (Appendix S3). Both VZ and ELZ were not aware of the intention of MVK to use their photographs for the present study until the end of the visit to Brazil, and therefore this data set (DS4) was presumed to be collected in a blind way. The fifth data set (DS5 hereafter) consisted of nature and wildlife photographs found in the WWW. It differs from DS4 by the wider coverage of localities in Brazil and of the timing of photographing.

**Table 1:** Data sets used in the analyses.

Data set	Data source	Selection of plants	Method of data collection	Sample size
DS1	Publications	Non-blind	From plants	35 publications containing 93 values of foliar losses to insects
DS2	Original field collected data	Non-blind	From plants	Foliar losses of 10 plant species
DS3	Photographs of plants from DS2	Non-blind	From images of plants	10 photographs
DS4	Various photographs by the authors (holiday snaps)	Blind	From images of plants	29 photographs
DS5	Various photographs found in the WWW	Blind	From images of plants	30 photographs

### Collection of published data

To be included in DS1, a published study had to be conducted in a way that assures the comparability of its results with the results of field measurements conducted by MVK, i.e., had to fit the following criteria:

(1) Losses of plant foliage, in terms of the proportion of damaged leaves, leaf area or leaf biomass consumed (or modified, as in the case of gallers) by herbivorous insects from three feeding guilds (defoliators, miners and gallers) were measured from individual leaves of woody plants and were reported in a form that allowed calculation of the mean values of these losses (i.e., median values and indices that could not be transformed to the percentages were excluded);
(2) The leaves for measurements of herbivory were selected in such a way that they were representative of the plant-wide level of damage (i.e., studies separately reporting the levels of damage of young and mature leaves were excluded), and the measured plant individuals were haphazardly selected from the population (i.e., studies separately reporting the levels of damage of healthy and damaged trees, resistant and susceptible trees or trees foraged and not foraged by ants were excluded);
(3) The study was conducted in a natural forest or savanna (cerrado) ecosystem (i.e., data collected from mangroves, orchards, plantations and urban plantings were excluded);
(4) The losses were measured in plant species that were native to the study region;
(5) The losses were measured for adult plants (i.e., seedlings and saplings were excluded);
(6) The losses to insect feeding were not combined with losses to fungal damage or to other damaging agents;
(7) The losses were attributable to the entire community of herbivorous insects (i.e., data on plant damage caused by a single insect species were excluded) at the background level of their density (i.e., losses caused by rare events of mass occurrence of a certain herbivore were excluded);
(8) The researchers did not mention specifically that losses were measured on young leaves soon after their flush.

We searched for publications that met these criteria in the ISI Web of Science database and Google Scholar using several conbinations of keywords (“Brazil” or “cerrado”, “folivor*” or “herbivor*”, “tree*” or “shrub*” or “woody”, “insect” and “damag*” or “loss*” or “consum*”) and further examined the reference lists of the identified papers. The search was completed on 31 October 2014.

When extracting the data from multiyear studies, we averaged values for all study years. If the study involved some manipulations, we used the data from non-treated (control) plants.

### Field measurements of foliar damage

To create DS2, MVK measured foliar damage in mid-September of 2013 in four areas of Brazil: around Ilhéus on the Atlantic coast of Bahia (14°48′S, 39°04′W), in Vale do Capão, Chapada Diamantina National Park in the inland region of Bahia (12°37′S, 41°30′W), near Novo Airão on the banks of the Rio Negro (2°38′S, 60°57′W) and in about the middle of the Trans-Pantanal road (16°25′S, 56°40′W). In each study area, MVK selected one to four abundant plant species and took their photographs for future identification (Appendix S2), which served the basis for DS3. He then haphazardly selected one branch (with about 100 leaves, from 1 to 3 m above ground) from each of two conspecific plant individuals, pointing at the selected branch from 5–10 m distance (i.e., from a distance that did not allow recognition of foliar damage imposed by insects).

Following widely used methodology (e.g., Southwood, Moran & Kennedy, 1982; Fox & Morrow, 1983; Alliende, 1989), each leaf from the selected branches was attributed to one of the following damage classes according to the proportion of the area of leaf lamina that was consumed or damaged by insects: intact leaves, 0.01–1, 1.01–5, 5.01–25, 25.01–50, 50.01–75 and 75.01–100 percent. This attribution was conducted four times for each leaf: separately for defoliators, miners and gallers, and for all these guilds combined. The numbers of leaves in each damage class were recorded separately for each feeding guild and for the total damage. From each individual plant, we calculated from these data (a) the proportions of leaves damaged by defoliators, miners, gallers and by all herbivores together (as the ratio between the number of damaged leaves and the total number of surveyed leaves, multiplied by 100%) and (b) the proportions of leaf area lost to (or damaged by) insects from each of these feeding guilds. The latter value was calculated as follows: the number of leaves in each damage class was multiplied by the respective median value of the damaged leaf area (i.e., 0 for intact leaves, 0.5% for the damage class 0.01–1%, 3% for the damage class 1.01–5%, etc.); the obtained values were summed across all damage classes and divided by the total number of leaves (including undamaged ones) within a sample (Alliende, 1989). Data obtained from two plant individuals were averaged prior the further analyses.

### Selection and processing of photographs

To be included in DS4, a photograph showing leaves of woody plants had to fit the following criteria:

(1) the photo was taken for the purposes other that studying of insect herbivory (i.e., holiday snaps);
(2) the photo was taken in natural or semi-natural forest or cerrado habitat;
(3) the branches of woody plants seen on the photo were not more than 3 m above the ground;
(4) the images were of sufficient quality to allow an accurate estimate of the proportion of leaf area consumed by defoliating insects.

The photographs that formed DS5 were searched in the WWW on 7 November 2014 using keywords “Brazil” and “nature” or “forest” or “birds” or “animals” in both English and Russian. To create a sample of about the same size as DS4 (that contains 29 photographs), we selected the first 30 photographs satisfying the criteria 1, 2 and 4 (coined above) and containing images of at least 10 leaves (or leaflets) that were sharp enough for the analysis of their damage from about 2,500 surveyed photographs. We rejected photographs showing leaves of palms, because palms were absent in DS1–DS4.

In all photographs, we visually examined images of all leaves in which at least a half of the leaf lamina was clearly visible, attributed each of these leaves (or leaf parts) to one of the damage classes, and calculated the proportion of leaves damaged by defoliators and the proportion of leaf area lost to these insects as described above, i.e., exactly in the same way as in field measurements of foliar damage. If only a part of the leaf lamina was visible, then the percent of damage was estimated relative to the area of the visible part of the leaf, not to the area of the entire leaf. The estimation of foliar damage in DS4 was independently conducted by three persons, two of which were not informed of the hypothesis being tested.

To exclude the possibility that DS4 was biased in an opposite direction, i.e., towards lower plant damage due to selection of aesthetically pleasing objects or background for photographing, we compared the total losses of leaf area among photographs showing individual plants (selected for photographing for their beauty), persons (who may be asked to move to another position if the background contains unsightly objects), landscapes and animals (photographed immediately after they were seen), as well as between photographs focused on plants and having plants as a part of the background.

### Data analysis

Distributions of both characteristics of herbivory (i.e., of proportions of damaged leaves and consumed leaf area) were greatly skewed and therefore were analysed by non-parametric methods. The reliability of the photograph-based estimates of foliar losses was checked by comparing (a) the results obtained by three different persons who evaluated the same set of photographs (within DS4), and (b) by comparing the measurements taken from plants (DS2) and from photographs of these plants (DS3). These comparisons were performed using signed rank test (SAS UNIVARIATE procedure; SAS Institute, 2009). The analysis of differences in foliar losses between different groups of photographs were evaluated by the Kruskal–Wallis test (SAS NPAR1WAY procedure; SAS Institute, 2009).

The prediction (1) that non-blindly collected data tends to overestimate the level of herbivory was tested by contrasting (with the Kruskal–Wallis test) these data (DS1 combined with DS2) with blindly collected data (DS4 combined with DS5 after the Kruskal–Wallis tests revealed no differences between them). The prediction (2) that the foliar losses calculated from published studies will be higher than those measured by one of the authors of the present study was tested by contrasting (with the Kruskal–Wallis test) the published data (DS1) and the original data (DS2).

The reporting and/or publication bias was searched for by calculating a Kendall *τ_B_* correlation coefficient between sample size and the reported loss to insects (SAS CORR procedure; SAS Institute, 2009). Following Jennions et al. (2013), we presumed that if such a bias acts against small-sample studies that demonstrated unexpectedly low levels of herbivory, then the reported foliar damage should decrease with the increase in sample size.

## Results

### Analysis of published data

We identified 42 papers (published from 1990 to 2014) fitting our search criteria. These papers (DS1) reported 94 values of the proportion of leaf area consumed or damaged by insects, and 23 values of the proportion of leaves damaged by insects (Appendix S1). Only two of the 42 papers separately reported foliar losses for all feeding guilds of insects (defoliators, miners and gallers) considered in our analysis. The researchers who studied 1–3 species of woody plants reported a two-fold larger consumption of their foliage by insects than did researchers who studied 10 or more plant species (mean ± S.E.: 8.30 ± 1.03% based on 34 studies and 3.84 ± 0.72% based on 7 studies, respectively; *χ*^2^ = 4.47, df = 1, *P* = 0.03).

The proportion of leaf area lost to all feeding guilds did not differ from the proportion of leaf area lost to externally feeding defoliators alone (Kruskal–Wallis test: *χ*^2^ = 0.07, df = 1, *P* = 0.80), justifying the combination of these data for the search of publication bias and demonstrating that miners and gallers usually contribute only a minor fraction to the total plant damage by herbivorous insects.

We did not find evidence for the existence of publication and/or reporting bias for the total values of foliar losses, combined with losses to defoliators: these values were independent of sample size (number of study trees: *τ_B_* = 0.07, *n* = 83, *P* = 0.38; number of measured leaves: *τ_B_* = 0.01, *n* = 66, *P* = 0.99). The shortage of published data did not allow performing a similar analysis for miners and gallers.

### Validation of methodology

For the ten plant species we studied, the foliar losses to defoliators estimated from non-blindly taken photographs (DS3) did not differ from the results of the field measurements (DS2; signed rank test, proportion of damaged leaves: *S* = 9.5, df = 8, *P* = 0.30; proportion of consumed leaf area: *S* = 11.5, df = 8, *P* = 0.20), thus confirming that the analysis of photographs provides reliable estimates of folivory.

The measurements made by three different persons from the same set of 29 original photographs of blindly selected plants (DS4; Appendix S3) yielded similar values for both the proportion of damaged leaves and the proportion of leaf area lost to defoliators (signed rank tests, all *P* > 0.20); therefore, we averaged the results of three measurements for each photograph prior to further analysis.

Absence of differences in losses of leaf area among photographs taken from individual plants, persons, landscapes and animals (Kruskal–Wallis test; *χ*^2^ = 0.76, df = 3, *P* = 0.86), as well as between photographs focused on plants and showing plants as a part of background (*χ*^2^ = 0.05, df = 1, *P* = 0.82) suggests that the level of foliar damage did not influence selection of the objects for photographing, i.e., DS4 can be considered as an unbiased sample in relation to foliar losses of woody plants to insects.

### Comparison between data sets

**Figure 1:**
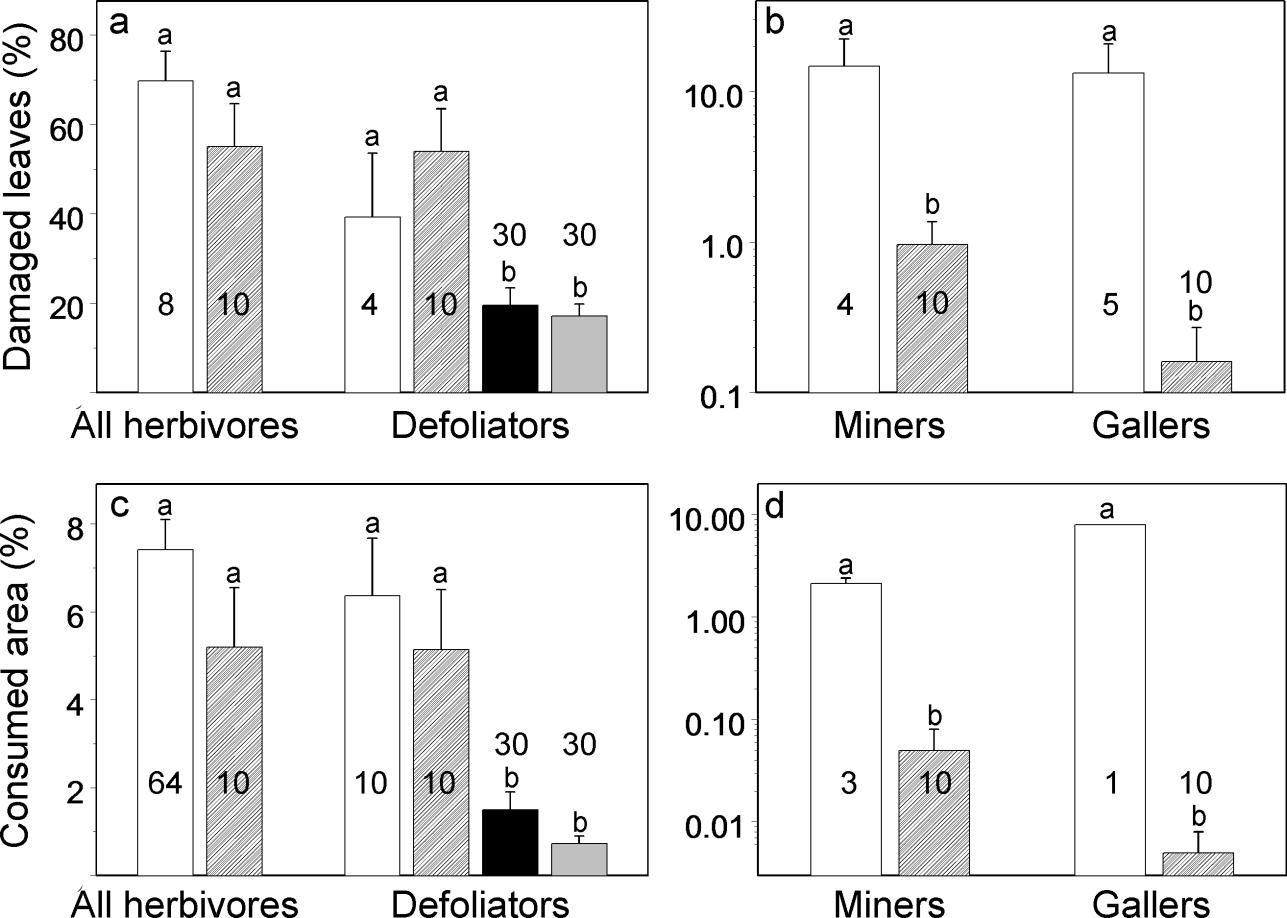
Comparisons between measurements of foliar damage of woody plants for total damage and separately for three feeding guilds of herbivorous insects. (A, B) proportion of damaged leaves; (C, D) consumed or damaged leaf area. Open bars: published data (DS1, non-blind); hatched bars: original data collected from plants (DS2, non-blind); black filled bars: data collected from original photographs of plants (DS4, blind); grey filled bars: data collected from photographs of plants found in the WWW (DS5, blind). Bars indicate standard errors; sample sizes (i.e., numbers of individual records) are shown in/above the bars. Within each group of herbivores, bars labelled with different letters differ from each other at the probability level *P* < 0.05; for the actual results of the statistical tests, see text.

The results of our non-blind field measurements (DS2) did not differ from published data (DS1) in terms of both total losses of woody plant foliage to insects and losses to externally feeding defoliators (Figs. 1A and 1C). However, we found significantly (15 to 1,600 times) lower damage by miners and gallers than the damage reported in published studies (Figs. 1B and 1D).

We found no differences between our original photographs (DS4) and photographs found in the WWW (DS5) in either proportion of damaged leaves (Fig. 1A; *χ*^2^ = 0.05, df = 1, *P* = 0.82) or losses of leaf area (Fig. 1C; *χ*^2^ = 2.75, df = 1, *P* = 0.10). Therefore the blindly collected DS4 and DS5 were combined for the further analysis and contrasted to non-blindly collected DS1 and DS2. The blindly collected data on losses of leaf area of woody plants to externally feeding defoliators (measured from photographs) were, on average, one-fifth of the losses obtained by means of non-blind field measurements (Fig. 1C; *χ*^2^ = 25.5, df = 1, *P* < 0.0001), and blindly collected data on the proportion of damaged leaves were, on average, one-third of values obtained by means of non-blind field measurements (Fig. 1A; *χ*^2^ = 10.5, df = 1, *P* = 0.0012).

## Discussion

Developing reliable methods that can be used to improve our understanding of ecological patterns and processes represents a key challenge for ecology (Bocedi et al., 2012). In this study we compared for the first time estimates of plant losses to defoliating insects obtained blindly with the data collected by traditional, non-blind methods. Data obtained from blindly taken photographs demonstrated that woody plants in Brazil lose, on average, 1.11% of their leaf area to defoliating insects. This value is more than ten times lower than the average values reported for tropical regions in several highly cited review papers (from 11.1 to 48.0%: Coley & Aide, 1991; Cyr & Pace, 1993; Coley & Barone, 1996). Thus, we confirmed our prediction (1) that non-blindly collected data tends to overestimate the community-wide levels of herbivory in the tropics.

We are sure that the low level of foliar losses obtained by a blind method cannot be attributed to methodological reasons. First, plant- and photograph-based measurements of foliar losses in ten plant species (i.e., DS2 and DS3) did not differ statistically from each other, confirming the validity of the data obtained from photographs. Second, the average proportion of leaf area lost to insects is primarily determined by the number of moderately and severely damaged leaves (over 5 percent of leaf area consumed), and these leaves are difficult to miss on a high-quality photograph. Of course, some slightly damaged leaves could be classified as intact on the basis of a photograph. However, if we classify all leaves placed in the lowest damage class (0.01 to 1 percent of leaf area consumed) in our field-collected data (DS2) as intact, then the average loss of leaf area decreases only slightly (from 5.14 to 5.06%). We also demonstrated that our blind method of assessing herbivore damage from photographs was unlikely to underestimate the species-specific values of herbivory due to selection of aesthetically pleasing objects (i.e., plants that show no visible signs of herbivory), because visibly damaged plants were not avoided when served as a background for other-purpose photographing.

Contrary to expectations (our prediction 2), we found no statistically significant differences between the published data (DS1) and our field measurements of plant losses to defoliating insects (DS2). First, this result hints at the absence of publication bias, a conclusion that is in line with the independence of the published values of plant losses from respective sample sizes. Second, this result suggests that the researcher’s selection of plant species for measurements of folivory is not influenced by the knowledge of the local environment. Third, the intention of the researcher (MVK) to select study species independently of the level of foliar damage appeared insufficient to avoid bias, thus confirming that this bias resulted from unconscious psychological processes.

Tree species in tropical forests differ considerably in the levels of herbivory they experience (Lowman, 1995; Rinker & Lowman, 2004), and it seems that both earlier researchers and MVK, while using a non-blind method, unconsciously avoided plant species that showed no or little foliar damage, i.e., were ‘not typical’ for a tropical region. The selection for plant species showing higher-than-average herbivore damage was demonstrated for both published and original records, because both estimates significantly exceeded those obtained in a blind test, i.e., from haphazardly taken photographs (DS4 and DS5). Although interpretation of photographs could also be influenced by the researcher’s expectancies, in the case of the person who was aware of the research hypothesis tested in our study, two other persons who were not informed about the research hypothesis showed similar results. Therefore, we conclude that our records made from photographs are not biased, and that obtaining an accurate community-wide estimate of the background herbivory is generally prevented by biased selection of plant species with higher-than-average levels of foliar damage. This selection may be partly due to confirmation bias caused by the influence of the dominating concept (e.g., Coley & Aide, 1991; Coley & Barone, 1996) suggesting high levels of herbivory in the tropics.

In some situations, preferential selection of plant species showing higher-than-average herbivore damage may be justified by the purpose of the study. For example, the researchers who aimed to explore the distribution of herbivory across the canopy (e.g., Nascimento, 1989; Ribeiro & Basset, 2007), or to compare herbivory on young and mature individuals (e.g., Oliveira et al., 2012), unavoidably selected plant species bearing visible signs of herbivory. However, the non-significant difference between our own records (aimed at estimating community-wide values of foliar losses) and the published data indicates that the influence of this factor on published values of plant damage by insects is relatively minor.

On the other hand, in the case of leaf miners and gallers, selection for highly damaged plant species for research purpose reasons is obviously more important than in the case of defoliators. Mining and galling insects at their background densities consume or damage a small proportion of leaves. Therefore, plant losses to miners and gallers are estimated mostly in studies specifically exploring these groups of insects, and only when their occurrence is well manifested. Damage by miners and gallers might also be underreported because the obtained values were rather small and therefore considered ‘negligible,’ some researchers (e.g., Oliveira et al., 2012) honestly mentioned that miners and/or gallers were rare and therefore their occurrence had not been measured. These two factors may explain why the difference between original (DS2) and published (DS1) values of woody plant damage by miners and gallers is so high (from 15 to 1,600 times!). A shortage of published information on plant losses to herbivores from these feeding guilds calls for collection of more data and for reporting these losses even when they approach zero or are equal to zero.

The striking (more than two-fold) difference between the outcomes of studies that explored insect damage in one to three plant species and in ten or more species suggests that the bias in studies addressing the levels of folivory can be reduced by increasing the number of investigated plant species. In particular, global foliar losses to herbivores (5.3%) appeared substantially lower than previously estimated when data from 1058 species of vascular plants were analysed (Turcotte et al., 2014). On the other hand, it is possible to choose study species randomly from the list of plants occurring in the study region, but this method seems impractical and we are not aware of any publication that employed this approach. Another way to avoid bias in selection of plant species is to perform a systematic sampling from all plant species within a set of randomly selected study plots. For example, Vasconcelos (1999), who used systematic sampling and explored around 80 species of canopy trees near Manaus in Brazil, reported that the average foliar loss of adult trees to insects was 2.90%. Similarly, Zava & Cianciaruso (2014) who sampled 983 individuals of 75 tree species from 49 plots in Emas National Park in Brazil reported that community-wide losses of woody plants to insects were as low as 1.35%, i.e., very close to our value (1.11%) obtained by a blind method.

To conclude, the mean level of background insect herbivory, estimated from published data on the damage of individual species of woody plants, is significantly higher than the community-wide loss of woody plant foliage to insects, estimated by a blind method. We did not find publication bias and therefore, on the basis of comparison between blind and non-blind records, we suggest that the main reason behind the overestimation of the community-wide level of folivory is the presence of confirmation bias in the form of selection bias. The magnitude of this bias in research on different types of terrestrial ecosystems requires further investigation, but our data suggest that we may need to revise the paradigm of the high level of background insect herbivory in tropical regions. Based on our findings, we urge for caution in obtaining community-wide values of herbivory and, potentially, of other ecosystem characters, from the results of multiple single-species studies.

In general, we unequivocally (i.e., by direct comparison between samples collected blind and non-blind) demonstrated for the first time the existence of confirmation bias in ecological studies and its effect on our knowledge. While medical, psychological and cognitive sciences long ago appreciated the influence of confirmation bias on the outcome of the research, this bias was overlooked in ecology. In particular, the potential impact of confirmation bias (and of other biases introduced by the researcher) on our understanding of ecological patterns and processes was not listed among the recently identified top 100 fundamental ecological questions, although the impact of publication bias was included in this list (Sutherland et al., 2013). Different kinds of biases may considerably influence ecological research, and therefore we hope that our study will attract the attention of ecologists to this problem and stimulate both the development and the wide application of methods that avoid biases occurring due to unconscious psychological processes.

## Supplemental Information

10.7717/peerj.709/supp-1 Appendix S1 Published data on foliar losses of woody plants to insects in Brazil.

10.7717/peerj.709/supp-2 Appendix S2 Foliar damage of woody plants measured in the field and from photographs of these plants. Photo credit: Vitali Zverev

10.7717/peerj.709/supp-3 Appendix S3 Foliar damage of woody plants measured from nature, wildlife and portrait photographs. Photo credit: Vitali Zverev and Elena Zverev

10.7717/peerj.709/supp-4 Appendix S4 Foliar damage of woody plants measured from nature and wildlife photographs found on the internet.
