# Supplementary material for: Confirmation bias leads to overestimation of losses of woody plant foliage to insect herbivores in tropical regions
Source: PeerJ. 2014 Dec 23;2:e709. doi: 10.7717/peerj.709 (PMC4277485; doi:10.7717/peerj.709)
Supplement: Appendix S1 [file peerj-02-709-s001.pdf]

Appendix S1. Published data on foliar losses of woody plants to insects in Brazil.

No	Continent	Latitude	Longitude	Altitude	Habitat	Family	Plant species
1	28-45 km N of Manaus	2.77 S	60.00 W	80	Secondary forest	.	COMMUNITY-WIDE
2	28-45 km N of Manaus	2.77 S	60.00 W	80	Primary rain forest	.	COMMUNITY-WIDE
3	70 km N Manaus	2.42 S	59.80 W	100	Primary rain forest	Melastomataceae	*Maieta guianensis*
4	70 km N Manaus	2.50 S	60.00 W	100	Primary rain forest	Melastomataceae	*Tococa bullifera*
5	Belo Horizonte	19.87 S	43.97 W	840	Cerrado	Bignoniaceae	*Tabebuia ochracea*
6	Belo Horizonte	19.87 S	43.97 W	840	Cerrado	Bignoniaceae	*Tabebuia aurea*
7	Belo Horizonte	19.87 S	43.97 W	840	Mesic forest	Bignoniaceae	*Tabebuia ochracea*
8	Belo Horizonte	19.87 S	43.97 W	840	Cerrado	Bignoniaceae	*Tabebuia ochracea*
9	Belo Horizonte	19.87 S	43.97 W	840	Cerrado	Bignoniaceae	*Tabebuia ochracea*
10	Belo Horizonte	19.87 S	43.97 W	840	Cerrado	Bignoniaceae	*Tabebuia aurea*
11	Belo Horizonte	19.87 S	43.97 W	840	Cerrado	Bignoniaceae	*Tabebuia aurea*
12	Cerrado Pe-de-Gigante	21.64 S	47.64 W	650	Cerrado	Apiaceae	*Didymopanax vinosum*
13	Cerrado Pe-de-Gigante	21.64 S	47.64 W	640	Grassland with scattered trees and shrubs	Apiaceae	*Didymopanax vinosum*
14	Cerrado Pe-de-Gigante	21.64 S	47.62 W	680	Tall woodland (cerrado)	Apiaceae	*Didymopanax vinosum*
15	Cerrado Pe-de-Gigante	21.64 S	47.64 W	650	Cerrado	Apiaceae	*Didymopanax vinosum*
16	Cerrado Pe-de-Gigante	21.64 S	47.64 W	640	Grassland with scattered trees and shrubs	Apiaceae	*Didymopanax vinosum*
17	Cerrado Pe-de-Gigante	21.64 S	47.62 W	680	Tall woodland (cerrado)	Apiaceae	*Didymopanax vinosum*
18	Cerrado research station	21.75 S	45.01 W	960	Cerrado	Vochysiaceae	*Qualea grandiflora*
19	Clube de Caca e Pesca Itororo	18.98 S	48.30 W	740	Cerrado	Ochnaceae	*Ouratea spectabilis*
20	Cuiaba	15.60 S	56.10 W	170	Cerrado	Vochysiaceae	*Vochysia rufa*
21	Cuiaba	15.60 S	56.10 W	170	Cerrado	Dilleniaceae	*Curatella americana*
22	Cuiaba	15.60 S	56.10 W	170	Cerrado	Vochysiaceae	*Vochysia rufa*
23	Cuiaba	15.60 S	56.10 W	170	Cerrado	Dilleniaceae	*Curatella americana*
24	Ecological Station Pirapitinga	18.34 S	45.31 W	570	Cerrado	Vochysiaceae	*Qualea parviflora*
25	Ecological Station Pirapitinga	18.34 S	45.31 W	570	Cerrado	Vochysiaceae	*Qualea parviflora*
26	Ecological Station Pirapitinga	18.34 S	45.31 W	570	Cerrado	Vochysiaceae	*Qualea parviflora*
27	Ecological Station Pirapitinga	18.34 S	45.31 W	570	Cerrado	Fabaceae	*Bauhinia brevipes*
28	Ecological Station Pirapitinga	18.34 S	45.31 W	570	Cerrado	Vochysiaceae	*Qualea parviflora*

29	Fazenda Agua Limpa	15.93 S	44.92 W	600	Cerrado	Erythroxylaceae	*Erythroxylum tortuosum*
30	Fazenda Agua Limpa	15.93 S	44.92 W	600	Cerrado	Malpighiaceae	*Byrsonima verbascifolia*
31	Fazenda Agua Limpa	15.93 S	44.92 W	600	Cerrado	Euphorbiaceae	*Maprounea guianensis*
32	Fazenda Agua Limpa	15.93 S	44.92 W	600	Cerrado	Erythroxylaceae	*Erythroxylum campestre*
33	Fazenda Agua Limpa	15.93 S	44.92 W	600	Cerrado	Clusiaceae	*Kielmeyera coriacea*
34	Fazenda Agua Limpa	15.93 S	44.92 W	600	Cerrado	Clusiaceae	*Kielmeyera variabilis*
35	Fazenda Agua Limpa	15.93 S	44.92 W	600	Cerrado	Rubiaceae	*Palicourea rigida*
36	Fazenda Agua Limpa	15.93 S	44.92 W	600	Cerrado	Malpighiaceae	*Peixotoa goyana*
37	Fazenda Agua Limpa	15.93 S	44.92 W	600	Cerrado	Lamiaceae	*Hyptis saxatilis*
38	Fazenda Agua Limpa	15.93 S	44.92 W	600	Cerrado	Malpighiaceae	*Byrsonima coccolobifolia*
39	Fazenda Agua Limpa	15.93 S	44.92 W	600	Cerrado	Erythroxylaceae	*Erythroxylum deciduum*
40	Fazenda Agua Limpa	15.93 S	44.92 W	600	Cerrado	Vochysiaceae	*Qualea grandiflora*
41	Fazenda Agua Limpa	15.93 S	44.92 W	600	Cerrado	Euphorbiaceae	*Croton goyazensis*
42	Fazenda Agua Limpa	15.93 S	44.92 W	600	Cerrado	Apocynaceae	*Aspidosperma tomentosum*
43	Fazenda Agua Limpa	15.93 S	44.92 W	600	Cerrado	Malpighiaceae	*Byrsonima crassa*
44	Fazenda Agua Limpa	15.93 S	44.92 W	600	Cerrado	Rubiaceae	*Chomelia ribesioides*
45	Fazenda Agua Limpa	15.93 S	44.92 W	600	Cerrado	Malvaceae	*Pavonia rosa-campestris*
46	Fazenda Agua Limpa	15.93 S	44.92 W	600	Cerrado	Rubiaceae	*Sabicea brasiliensis*
47	Fazenda Agua Limpa	15.93 S	44.92 W	600	Cerrado	Vochysiaceae	*Qualea parviflora*
48	Fazenda Agua Limpa	15.93 S	44.92 W	600	Cerrado	Erythroxylaceae	*Erythroxylum suberosum*
49	Fazenda Jatoba	13.88 S	45.70 W	760	Cerrado	Melastomataceae	*Tococa guainanensis*
50	Gloria's Farm	18.95 S	48.20 W	920	Cerrado	Solanaceae	*Solanum lycocarpum*
51	Maraca Island	3.42 N	61.67 W	150	Forests	Poaceae	*Pradosia surinamensis*
52	Maraca Island	3.42 N	61.67 W	150	Forests	Sapotaceae	*Ecclinusa guianensis*
53	Maraca Island	3.42 N	61.67 W	150	Forests	Fabaceae	*Peltogyne gracilipes*
54	Mata Seca State Park	14.88 S	43.99 W	460	Seasonally dry tropical forest	Bignoniaceae	*Handroanthus spongiosus*
55	Mata Seca State Park	14.88 S	43.99 W	460	Seasonally dry tropical forest	Bignoniaceae	*Handroanthus spongiosus*
56	Municipality of Montes Claros	16.75 S	43.92 W	880	Savanna	Caryocaraceae	*Caryocar brasiliense*
57	Pantanal do rio Miranda	21.47 S	56.15 W	290	Pantanal	Bignoniaceae	*Tabebuia aurea*
58	Pantanal do rio Miranda	21.47 S	56.15 W	290	Pantanal	Bignoniaceae	*Tabebuia aurea*
59	Pantanal do rio Miranda	21.47 S	56.15 W	290	Pantanal	Bignoniaceae	*Tabebuia aurea*

60	Parnamirim	8.08 S	39.57 W	230	Dry tropical forest	Euphorbiaceae	*Cnidoscolus quercifolius*
61	Piloes Valley	23.90 S	46.50 W	210	.	Melastomataceae	*Tibouchina pulchra*
62	Piloes Valley	23.90 S	46.50 W	210	.	Melastomataceae	*Tibouchina pulchra*
63	Rio Pandeiros	15.60 S	44.67 W	620	Cerrado = savanna	.	COMMUNITY-WIDE
64	Rio Pandeiros	15.60 S	44.67 W	620	Dry forests	.	COMMUNITY-WIDE
65	Saint-Hilaire Forest	16.60 S	49.26 W	800	Mesic semi-deciduous forest	Styracaceae	*Styrax pohlii*
66	Sao Carlos	21.98 S	47.86 W	850	Cerrado	Verbenaceae	*Aegiphila lhotzkiana*
67	Sao Carlos	21.98 S	47.86 W	850	Cerrado	Ebenaceae	*Diospyros hispida*
68	Sao Carlos	21.98 S	47.86 W	850	Cerrado	Fabaceae	*Stryphnodendron adstringens*
69	Sao Carlos	21.98 S	47.86 W	850	Cerrado	Styracaceae	*Styrax ferrugineus*
70	Sao Carlos	21.98 S	47.86 W	850	Cerrado	Bombacaceae	*Eriotheca gracilipes*
71	Sao Carlos	21.98 S	47.86 W	850	Cerrado	Asteraceae	*Vernonia* sp.
72	Sao Carlos	21.98 S	47.86 W	850	Cerrado	Fabaceae	*Stryphnodendron polyphyllum*
73	Sao Carlos	21.98 S	47.86 W	850	Cerrado	Asteraceae	*Piptocarpha rotundifolia*
74	Sao Carlos	21.98 S	47.86 W	850	Cerrado	Rubiaceae	*Rourea induta*
75	Sao Carlos	21.98 S	47.86 W	850	Cerrado	Bignoniaceae	*Tabebuia ochracea*
76	Sao Carlos	21.98 S	47.86 W	850	Cerrado	Connaraceae	*Connarus suberosus*
77	Saraca-Taquera National Forest	1.40 S	56.27 W	60	Evergreen equatorial moist forest	Anacardiaceae	*Tapirira guianensis*
78	Saraca-Taquera National Forest	1.40 S	56.27 W	60	Evergreen equatorial moist forest	Anacardiaceae	*Tapirira guianensis*
79	Uberlandia	18.95 S	48.20 W	950	Cerrado	Vochysiaceae	*Qualea multiflora*
80	Uberlandia	18.95 S	48.20 W	950	Cerrado	Lythraceae	*Lafoensia pacari*
81	Uberlandia	18.95 S	48.20 W	950	Cerrado	Vochysiaceae	*Qualea multiflora*
82	Mata Seca State Park	14.88 S	43.99 W	460	Seasonally dry tropical forest	.	COMMUNITY-WIDE
83	Mata Seca State Park	14.88 S	43.99 W	460	Seasonally dry tropical forest	.	COMMUNITY-WIDE
84	Mata Seca State Park	14.88 S	43.99 W	460	Seasonally dry tropical forest	.	COMMUNITY-WIDE

85	Municipality of Montes Claros	16.75 S	43.92 W	880	Cerrado	Caryocaraceae	*Caryocar brasiliense*
86	Municipality of Montes Claros	16.75 S	43.92 W	880	Cerrado	Caryocaraceae	*Caryocar brasiliense*
87	Municipality of Montes Claros	16.75 S	43.92 W	880	Cerrado	Caryocaraceae	*Caryocar brasiliense*
88	Municipality of Montes Claros	16.75 S	43.92 W	880	Cerrado	Caryocaraceae	*Caryocar brasiliense*
89	Municipality of Montes Claros	16.75 S	43.92 W	880	Cerrado	Caryocaraceae	*Caryocar brasiliense*
90	Parque Estadual Intervales	24.27 S	48.40 W	850	Atlantic forest	Piperaceae	*Piper hispidum*
91	Serra do Cipo	19.25 S	43.58 W	100	Cerrado	Solanaceae	*Solanum lycocarpum*
92	Parque Municipal da Lagoa do Peri	27.72 S	48.54 W	3	Restinga	Myrtaceae	*Gomigesia palustris*
93	Parque Municipal da Lagoa do Peri	27.72 S	48.54 W	3	Restinga	Myrtaceae	*Gomigesia palustris*
94	Serra do Cipo	19.25 S	43.58 W	100	Cerrado		*Chamaecrista semaphora*
95	Emas National Park	18.64 S	52.91 W	800	Cerrado	.	COMMUNITY-WIDE
96	Fazenda Remanso	14.80 S	52.64 W	350	Forest	Fabaceae	*Peltogyne conferti*
97	Fazenda Remanso	14.80 S	52.64 W	350	Cerrado	Fabaceae	*Peltogyne conferti*
98	Duas Bocas	20.27 S	40.67 W	800	.	Euphorbiaceae	*Croton* sp.
99	Duas Bocas	20.27 S	40.67 W	800	.	Solanaceae	*Solanum hexandrum*
100	EFLEX	19.33 S	44.33 W	740	Savanna	Clusiaceae	*Kielmeyera coriacea*
101	BDFFP site	2.42 S	59.84 W	120	Rain forest	Melastomataceae	*Maieta guianensis*
102	BDFFP site	2.42 S	59.84 W	120	Rain forest	Melastomataceae	*Tococa bullifera*

Continued.

**No**	**Herbivores**	**No of sites**	**No of trees per site**	**No of leaves**	**Leaves damaged (%)**	**Leaf area consumed (%)**	**Reference**	**Data extracted from**	**Comments**
1	all	1	60	300	72.0	2.60	Vasconcelos 1999	Text p. 618, Tab. 1	
2	all	1	60	300	74.1	2.90	Vasconcelos 1999	Text p. 618, Tab. 1	
3	all	1	15	120	.	11.20	Vasconselos 1991	Fig. 2	
4	all	1	20	120	.	28.70	Bruna et al. 2004	Fig. 2b	With ants
5	miners	3	44	1672	.	1.60	Ribeiro & Brown 1999	Fig. 1	Leaf cohorts averaged

6	miners	3	27	837	.	2.50	Ribeiro & Brown 1999	Fig. 1	Leaf cohorts averaged
7	all	1	1	283	.	4.05	Ribeiro et al. 1994	Text p. 305	Years averaged
8	chewers	3	44	1672	.	10.80	Ribeiro & Brown 1999	Fig. 1	Leaf cohorts averaged
9	all	3	44	1672	.	12.40	Ribeiro & Brown 1999	Fig. 1	Leaf cohorts averaged
10	chewers	3	27	837	.	17.50	Ribeiro & Brown 1999	Fig. 1	Leaf cohorts averaged
11	all	3	27	837	.	20.00	Ribeiro & Brown 1999	Fig. 1	Leaf cohorts averaged
12	chewers	1	10	.	11.6	0.04	Varanda & Pais 2006	Tab. 1	
13	chewers	1	10	.	5.4	0.19	Varanda & Pais 2006	Tab. 1	
14	chewers	1	10	.	34.5	0.74	Varanda & Pais 2006	Tab. 1	
15	all	1	10	.	.	1.32	Varanda & Pais 2006	Tab. 1	
16	all	1	10	.	.	7.33	Varanda & Pais 2006	Tab. 1	
17	all	1	10	.	.	8.49	Varanda & Pais 2006	Tab. 1	
18	all	1	15	150	.	19.17	Costa *et al*. 1992	Fig. 1	May, control trees
19	all	1	11	99	.	4.20	Byk & Del-Claro 2010	Fig. 2	Control
20	all	1	3	193	55.5	2.92	Nascimento *et al*. 1990	Fig. 3	4 dates averaged
21	all	1	3	132	78.8	4.55	Nascimento *et al*. 1990	Fig. 3	4 dates averaged
22	all	1	3	47	76.0	10.50	Nascimento *et al*. 1990	Fig. 4	Last census - May 1986
23	all	1	3	30	94.0	18.25	Nascimento *et al*. 1990	Fig. 3	Last census - May 1986
24	all	3	15	60	.	5.71	Goncalves-Alvim *et al*. 2010	Tab. 1	Three sites averaged
25	all	1	30	600	77.1	6.05	Goncalves-Alvim *et al*. 2004	Tab. 5	
26	all	3	30	600	.	8.07	Goncalves-Alvim *et al*. 2011	Fig. 1	Mature leaves
27	all	1	170	3400	30.3	13.70	Cornelissen & Fernandes 2001	Fig. 2	
28	gallers	1	30	600	0.8	.	Goncalves-Alvim *et al*. 2004	Tab. 5	
29	all	1	5	25	.	3.00	Marquis *et al*. 2001	Fig. 3a	
30	all	1	5	25	.	3.70	Marquis *et al*. 2001	Fig. 3a	
31	all	1	5	25	.	4.10	Marquis *et al*. 2001	Fig. 3a	
32	all	1	5	25	.	4.40	Marquis *et al*. 2001	Fig. 3a	
33	all	1	5	25	.	4.70	Marquis *et al*. 2001	Fig. 3a	
34	all	1	5	25	.	4.70	Marquis *et al*. 2001	Fig. 3a	
35	all	1	5	25	.	4.90	Marquis *et al*. 2001	Fig. 3a	
36	all	1	5	25	.	5.10	Marquis *et al*. 2001	Fig. 3a	
37	all	1	5	25	.	5.20	Marquis *et al*. 2001	Fig. 3a	
38	all	1	5	25	.	6.80	Marquis *et al*. 2001	Fig. 3a	

39	all	1	5	25	.	7.00	Marquis *et al*. 2001	Fig. 3a	
40	all	1	5	25	.	7.00	Marquis *et al*. 2001	Fig. 3a	
41	all	1	5	25	.	7.70	Marquis *et al*. 2001	Fig. 3a	
42	all	1	5	25	.	8.80	Marquis *et al*. 2001	Fig. 3a	
43	all	1	5	25	.	9.00	Marquis *et al*. 2001	Fig. 3a	
44	all	1	5	25	.	9.20	Marquis *et al*. 2001	Fig. 3a	
45	all	1	5	25	.	10.3	Marquis *et al*. 2001	Fig. 3a	
46	all	1	5	25	.	11.80	Marquis *et al*. 2001	Fig. 3a	
47	all	1	5	25	.	12.30	Marquis *et al*. 2001	Fig. 3a	
48	all	1	5	25	.	13.00	Marquis *et al*. 2001	Fig. 3a	
49	chewers	1	30	30	.	5.93	Bizerri & Vieira 2002	Fig. 3	Old leaves
50	all	1	24	480	.	8.55	Moreira & Del-Claro 2005	Fig. 3	Control plants; two years averaged
51	all	2			.	6.1	Nascimento & Proctor 2001	Tab. 1	
52	all	2	.	.	.	11.9	Nascimento & Proctor 2001	Tab. 1	
53	all	2	.	.	.	14.0	Nascimento & Proctor 2001	Tab. 1	
54	all	1	5	45	.	7.40	Silva *et al*. 2012	Fig. 5a	April, late successional forest
55	chewers	1	20	100	.	12.80	Oliveira *et al*. 2012	Fig. 3	April data
56	all	1	25	400	.	0.95	Leite *et al*. 2012	Tab. 2	Autumn
57	miners	1	15	465	.	2.30	Ribeiro & Brown 1999	Fig. 1	Leaf cohorts averaged
58	chewers	1	15	465	.	8.30	Ribeiro & Brown 1999	Fig. 1	Leaf cohorts averaged
59	all	1	15	465	.	10.60	Ribeiro & Brown 1999	Fig. 1	Leaf cohorts averaged
60	chewers	4	80	400	.	10.68	Coelho *et al*. 2012	Text p. 455	Habitats averaged
61	all	1	40	1600	.	1.15	Furlan *et al*. 2004	Tab. 2	Two years averaged
62	gallers	1	40	1600	4.3	.	Furlan *et al*. 2004	Tab. 2	Two years averaged
63	all	1	.	.	.	1.95	Neves *et al*. 2010	Fig. 2a	
64	all	1	.	.	.	6.60	Neves *et al*. 2010	Fig. 2a	
65	gallers	1	30	.	22.8	.	Araújo *et al*. 2011	Text p. 1593	Two habitats averaged
66	all	1	6	.	.	0.00	Gadotti & Batalha 2010	Tab. 2	Control
67	all	1	9	.	.	0.90	Gadotti & Batalha 2010	Tab. 2	Control
68	all	1	18	.	.	1.80	Gadotti & Batalha 2010	Tab. 2	Control
69	all	1	4	.	.	1.80	Gadotti & Batalha 2010	Tab. 2	Control

70	all	1	13	.	.	2.20	Gadotti & Batalha 2010	Tab. 2	Control
71	all	1	2	.	.	2.60	Gadotti & Batalha 2010	Tab. 2	Control
72	all	1	30	.	.	2.80	Gadotti & Batalha 2010	Tab. 2	Control
73	all	1	2	.	.	2.90	Gadotti & Batalha 2010	Tab. 2	Control
74	all	1	2	.	.	3.90	Gadotti & Batalha 2010	Tab. 2	Control
75	all	1	5	.	.	6.80	Gadotti & Batalha 2010	Tab. 2	Control
76	all	1	1	.	.	12.30	Gadotti & Batalha 2010	Tab. 2	Control
77	chewers	1	30	150	.	2.81	Fernandes *et al*. 2012	Fig. 2	Two years averaged
78	gallers	1	30	150	.	7.94	Fernandes *et al*. 2012	Fig. 2	Two years averaged
79	all	1	15	300	.	2.40	Del-Claro *et al*.1996	Fig 1	Controls for 2 dates averaged
80	all	1	23	207	.	10.40	Korndorfer & Del-Claro 2006	Fig. 1a	Control plants
81	all	1	15	180	.	19.83	Nahas *et al*. 2012	Pers. comm.	Control group, last census (May 2010)
82	chewers	5	20	2790	.	5.16	Neves *et al*. 2014	Fig. 5	Late successional stage
83	miners	5	20	2790	35.5	.	Neves *et al*. 2014	Fig. 5	Late successional stage
84	gallers	5	20	2790	0.5	.	Neves *et al*. 2014	Text p. 17	All stages averaged
85	chewers	1	27	1296	.	4.94	Leite *et al*. 2013	Tab. 1	Four seasons averaged
86	miners	1	27	1296	.	0.10	Leite *et al*. 2013	Tab. 1	Four seasons averaged
87	gallers	1	27	1296	37.9	7.18	Leite *et al*. 2013	Tab. 2	Four seasons averaged
88	chewers	1	12	576	.	3.45	Leite *et al*. 2012	Tab. 3	Cerrado only
89	miners	1	12	576	15.9	.	Leite *et al*. 2012	Tab. 3	Cerrado only
90	chewers	1	20	682	57.8	9.00	Mello 2007	Fig. 2, text p. 4	
91	chewers	1	15	750	.	10.90	Cuevas-Reyes *et al*. 2013	Text p. 559	Rural site only
92	miners	1	15	225	7.1	.	Tito *et al*. 2012	Text p. 14	Shrubby restinga
93	miners	1	15	225	0.4	.	Tito *et al*. 2012	Text p. 14	Arboreal restinga
94	chewers	1	15	450	26.4	0.40	da Costa *et al*. 2013	Fig. 1	
95	all	49	20	4815	.	1.35	Zava & Cianciaruso 2014	Text p. 628	
96	all	1	16	80	.	15.10	Francoso *et al*. 2013	Text p. 224	
97	all	1	15	75	.	5.00	Francoso *et al*. 2013	Text p. 224	
98	chewers	1	8	32	100.0	3.45	Eutrópio & Silva 2009	Tab. 1	Old leaves only
99	all	.	10	20	.	7.66	Krohling *et al*. 2010	Tab. 1	Mature leaves only
100	all	1	20	100	.	1.11	Loyola & Fernandes 1993	Tab. 5	Mature leaves

101	all	1	282	2000	.	4.87	Vasconselos & Davidson 2000	Text p. 104	Weighed mean
102	all	1	47	350	.	10.32	Vasconselos & Davidson 2000	Text p. 104	Weighed mean

References

Araújo S.W., Julião G.R., Ribeiro B.A., Silva I.P.A. & Santos B.B. (2011). Diversity of galling insects in *Styrax pohlii* (Styracaceae): edge effect and use as bioindicators. *Revista de Biologia Tropical,* 59, 1589-1597.

Bizerril M.X.A. & Vieira E.M. (2002). *Azteca* ants as antiherbivore agents of *Tococa formicaria* (Melastomataceae) in Brazilian Cerrado. *Studies on Neotropical Fauna and Environment,* 37, 145-149.

Bruna E.M., Lapola D.M. & Vasconcelos H.L. (2004). Interspecific variation in the defensive responses of obligate plant-ants: experimental tests and consequences for herbivory. *Oecologia,* 138, 558-565.

Byk J. & Del-Claro K. (2010). Nectar- and pollen-gathering *Cephalotes* ants provide no protection against herbivory: a new manipulative experiment to test ant protective capabilities. *Acta Ethologica,* 13, 33-38.

Coelho M.S., Belmiro M.S., Santos J.C. & Fernandes G.W. (2012). Herbivory among habitats on the neotropical tree *Cnidoscolus quercifolius* Pohl. in a seasonally deciduous forest. *Brazilian Journal of Biology,* 72, 453-457.

Cornelissen T.G. & Fernandes G.W. (2001). Defence, growth and nutrient allocation in the tropical shrub *Bauhinia brevipes* (Leguminosae). *Austral Ecology,* 26, 246-253.

Costa F.M.C.B., Oliveira A.T. & Oliveira P.S. (1992). The role of extrafloral nectaries in *Qualea grandiflora* (Vochysiaceae) in limiting herbivory: an experiment of ant protection in cerrado vegetation. *Ecological Entomology,* 17, 363-365.

Cuevas-Reyes P., Gilberti L., González-Rodríguez A. & Fernandes G.W. (2013). Patterns of herbivory and fluctuating asymmetry in *Solanum lycocarpum* St. Hill (Solanaceae) along an urban gradient in Brazil. *Ecological Indicators,* 24, 557-561.

da Costa F.V., de Azevedo I.F.P., Braga L.D., Perillo L.N., Neves F.D., Leite L.O., da Silva B.L.R., Ribeiro L.C., Fernandes G.W. & Cuevas-Reyes P. (2013). Fluctuating asymmetry and herbivory in two ontogenetical stages of *Chamaecrista semaphora* in restored and natural environments. *Journal of Plant Interactions,* 8, 179-186.

Del-Claro K., Berto V. & Réu W. (1996). Effect of herbivore deterrence by ants on the fruit set of an extrafloral nectary plant, *Qualea multiflora* (Vochysiaceae). *Journal of Tropical Ecology,* 12, 887-892.

Eutrópio F.J. & Silva A.G. (2009). Comparative analysis of leaf area loss by herbivory in *Croton* sp. at the REBIO of Duas Bocas, Cariacica, Espírito Santo, Southeastern Brazil. *Natureza on line,* 7, 27-30.

Fernandes G.W., Santos J.C. & Gomes V.M. (2012). Misleading herbivory in a tropical tree. *Arthropod-Plant Interactions,* 6, 649-654.

Françoso R.D., Goeller A.J. & Lenza E. (2013). Herbivory and leaf mass in *Peltogyne confertiflora* (Mart. ex Hayne) Benth. Fabales Bromhead Fabaceae Lindl. at forest and cerrado. *Ecología Austral,* 23, 222-226.

Furlan C.M., Salatino A. & Domingos M. (2004). Influence of air pollution on leaf chemistry, herbivore feeding and gall frequency on *Tibouchina pulchra* leaves in Cubatão (Brazil). *Biochemical Systematics and Ecology,* 32, 253-263.

Gadotti C.A. & Batalha M.A. (2010). The 'Herbivory Uncertainty Principle': application in a cerrado site. *Brazilian Journal of Biology,* 70, 279-282.

Gonçalves-Alvim S.J., Collevatti R.G. & Fernandes G.W. (2004). Effects of genetic variability and habitat of *Qualea parviflora* (Vochysiaceae) on herbivory by free-feeding and gall-forming insects. *Annals of Botany,* 94, 259-268.

Gonçalves-Alvim S.J., Lana T.C., Ranieri B.D., Silveira F.A.O., Ribeiro V. & Fernandes G.W. (2010). Growth, defense and herbivory on young leaves of *Qualea parviflora* (Vochysiaceae) in three different cerrado habitats. *Neotropical Biology and Conservation,* 5, 86-92.

Gonçalves-Alvim S.J., Lana T.C., Ranieri B.D. & Fernandes G.W. (2011). Test of hypotheses about herbivory and chemical defences of *Qualea parviflora* (Vochysiaceae) in Brazilian cerrado. *Revista Brasileira de Botanica,* 34, 223-230.

Korndörfer A.P. & Del-Claro K. (2006). Ant defense versus induced defense in *Lafoensia pacari* (Lythraceae), a myrmecophilous tree of the Brazilian cerrado. *Biotropica,* 38, 786-788.

Krohling C.A., Eutrópio F.J. & Silva A.G. (2010). Insect-plant interactions in *Solanum hexandrum* Vell. (Solanaceae): pollination and herbivory in the Biological Reserve of Duas Bocas, Cariacica, Espírito Santo. *Natureza on line,* 8, 78-85.

Leite G.L.D., Veloso R.V., Zanuncio J.C., Almeida C.I.M.E., Ferreira P.S.F., Fernandes G.W. & Soares M.A. (2012). Habitat complexity and *Caryocar brasiliense* herbivores (Insecta: Arachnida: Araneae). *Florida Entomologist,* 95, 819-830.

Leite G.L.D., Veloso R.V., Zanuncio J.C., Fernandes G.W., Almeida C.I.M., Pereira J.M.M., Serrão J.E. & Soares M.A. (2013). Seasonal abundance of galling Insects (Hymenoptera) on *Caryocar brasiliense* (Malpighiales: Caryocaraceae) trees in the cerrado. *Florida Entomologist,* 96, 797-809.

Leite G.L.O., Veloso R.V., Zanuncio J.C., Almeida C.I.M., Ferreira P.S.F., Serrão J.E. & Ramalho F.S. (2012). Seasonal damage caused by herbivorous insects on *Caryocar brasiliense* (Caryocaraceae) trees in the Brazilian savanna. *Revista Colombiana de Entomología,* 38, 108-113.

Loyola R.Jr. & Fernandes G.W. (1993). Herbivory on *Kielmeyra coriacea* (Guttiferae): the effects of plant age, leaf development and quality. *Revista Brasileira de Biologia*, 53, 295-304.

Marquis R.J., Diniz I.R. & Morais H.C. (2001). Patterns and correlates of interspecific variation in foliar insect herbivory and pathogen attack in Brazilian cerrado. *Journal of Tropical Ecology,* 17, 127-148.

Mello M.A.R. (2007). Influence of herbivore attack patterns on reproductive success of the shrub *Piper hispidum* (Piperaceae). *Ecotropica,* 13, 1-6.

Moreira V.S.S. & Del-Claro K. (2005). The outcomes of an ant-treehopper association on *Solanum lycocarpum* St. Hill: increased membracid fecundity and reduced damage by chewing herbivores. *Neotropical Entomology,* 34, 881-887.

Nahas L., Gonzaga M.O. & Del-Claro K. (2012). Emergent impacts of ant and spider interactions: Herbivory reduction in a tropical savanna tree. *Biotropica,* 44, 498-505.

Nascimento M.T. & Proctor J. (2001). Leaf herbivory on three tree species in a monodominant and two other terra firme forests on Maracá Island, Brazil. *Acta Amazonica,* 31, 1-12.

Nascimento M.T., Villela D.M. & De Lacerda L.D. (1990). Foliar growth, longevity and herbivory in two "cerado" species near Cuiaba, MT, Brazil. *Revista Brasileira de Botanica*, 13, 27-32.

Neves F.S., Araújo L.S., Espírito-Santo M.M., Fagundes M., Fernandes G.W., Sanchez-Azofeifa G.A. & Quesada M. (2010). Canopy herbivory and insect herbivore diversity in a dry forest-savanna transition in Brazil. *Biotropica,* 42, 112-118.

Neves F.S., Silva J.O., Espírito-Santo M.M. & Fernandes G.W. (2014). Insect herbivores and leaf damage along successional and vertical gradients in a tropical dry forest. *Biotropica*, 46, 14-24.
Oliveira K.N., Espírito-Santo M.M., Silva J.O. & Melo G.A. (2012). Ontogenetic and temporal variations in herbivory and defense of *Handroanthus spongiosus* (Bignoniaceae) in a Brazilian tropical dry forest. *Environmental Entomology*, 41, 541-550.
Ribeiro S.P. & Brown V.K. (1999). Insect herbivory in tree crowns of *Tabebuia aurea* and *T. ochracea* (Bignoniaceae) in Brazil: contrasting the cerrado with the "Pantanal Matogrossense". *Selbyana*, 20, 159-170.
Ribeiro S.P., Pimenta H.R. & Fernandes G.W. (1994). Herbivory by chewing and sucking insects on *Tabebuia ochracea*. *Biotropica*, 26, 302-307.
Silva J.O., Espírito-Santo M.M. & Melo G.A. (2012). Herbivory on *Handroanthus ochraceus* (Bignoniaceae) along a successional gradient in a tropical dry forest. *Arthropod-Plant Interactions*, 6, 45-57.
Tito R., Geisler G.E., de Souza L.P., Castellani T.T. & Lopes B.C. (2012). Herbivory in *Gomidesia palustris* (Myrtaceae) in two formations of restinga, southern Brazil. In: *Association for Tropical Biology and Conservation. 49th annual meeting. Bonito, Mato Grosso de Sul, Brazil. Abstracts*, pp. 817.
Varanda E.M. & Pais M.P. (2006). Insect folivory in *Didymopanax vinosum* (Apiaceae) in a vegetation mosaic of Brazilian cerrado. *Brazilian Journal of Biology*, 66, 671-680.
Vasconcelos H.L. (1991). Mutualism between *Maieta guianensis* Aubl., a myrmecophytic melastome, and one of its ant inhabitants - ant protection against insect herbivores. *Oecologia*, 87, 295-298.
Vasconcelos H.L. (1999). Levels of leaf herbivory in Amazonian trees from different stages in forest regeneration. *Acta Amazonica*, 29, 615-623.
Vasconcelos H.L. & Davidson D.W. (2000). Relationship between plant size and ant associates in two Amazonian ant-plants. *Biotropica*, 32, 100-111.
Zava P.C. & Cianciaruso M.V. (2014). Can we use plant traits and soil characteristics to predict leaf damage in savanna woody species? *Plant Ecology*, 215, 625-637.
