# Supplementary material for: Confirmation bias leads to overestimation of losses of woody plant foliage to insect herbivores in tropical regions
Source: PeerJ. 2014 Dec 23;2:e709. doi: 10.7717/peerj.709 (PMC4277485; doi:10.7717/peerj.709)
Supplement: Appendix S2 [file peerj-02-709-s002.pdf]

Appendix 2. Foliar damage of woody plants measured in the field and from photographs of these plants[1].

Photo #	Locality	Habitat	Plant species[2]	Feeding guild	Field measurements[3]			Measurements taken from photographs[4]		
					n	dam	cons	n	dam	cons
105	Ilhéus	Atlantic forest	Melastomataceae, *Henriettella* sp.	chewers	167	80.0	5.76	81	40.7	1.06
				gallers	167	0	0			
				miners	167	0	0			
107	Ilhéus	Atlantic forest	Moraceae or Sapotaceae	chewers	219	11.9	0.22	166	13.2	0.36
				gallers	219	0	0			
				miners	219	0	0			
125	Ilhéus	Atlantic forest		chewers	166	41.8	2.83	56	44.6	4.09
				gallers	166	0.7	0.02			
				miners	166	3.7	0.11			
130	Diamantina	Subalpine shrubland	Asteraceae, *Vernonanthura* sp. (?)	chewers	243	79.1	8.68	118	59.3	4.95
				gallers	243	0	0			
				miners	243	1.9	0.06			
163	Diamantina	Subalpine shrubland	Fabaceae, Caesalpinioideae	chewers	239	51.2	4.10	245	24.4	1.73
				gallers	239	0	0			
				miners	239	0.4	0.01			
164	Diamantina	Subalpine shrubland	Asteraceae	chewers	185	14.2	0.69	151	14.6	0.77
				gallers	185	0	0			
				miners	185	1.8	0.26			
166	Diamantina	Subalpine shrubland	Lythraceae (?)	chewers	227	60.9	8.28	-[5]	-	-
				gallers	227	0	0			
				miners	227	0	0			
283	Novo Airão	Riverside	Fabaceae, Mimosoideae	chewers	168	94.7	5.74	151	32.4	1.33

[1] High-resolution images available from authors upon request.
[2] Identified from a photograph by K. Ruokalainen.
[3] Mean values based on measurements of two plant individuals (see text for details). n, total number of leaves; dam, proportion of leaves damaged by defoliating insects (%); cons, proportion of leaf area consumed by defoliating insects (%).
[4] Measurement is based on a photograph taken from one of two study individuals.
[5] The quality of the photograph is insufficient for measurements of foliar damage.

				gallers	168	0	0			
				miners	168	0	0			
471	Pantanal	Riverside	Combretaceae, *Combretum* sp.	chewers	213	78.8	14.02	146	56.2	4.12
				gallers	213	0.9	0.03			
				miners	213	1.9	0.02			
472	Pantanal	Riverside	Fabaceae, *Bauhinia* sp.	chewers	201	21.4	1.07	9	55.6	3.78
				gallers	201	0	0			
				miners	201	0	0			

Photographs

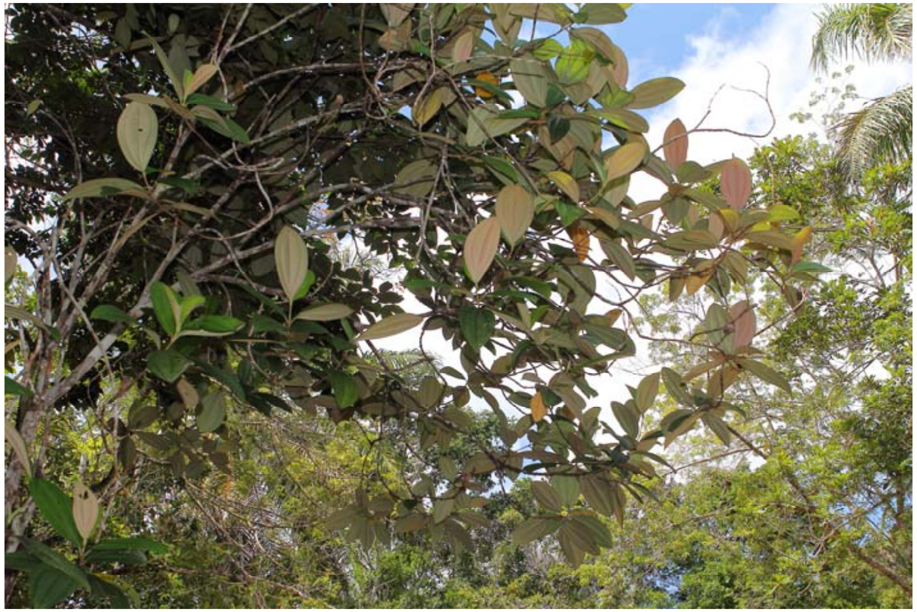

105

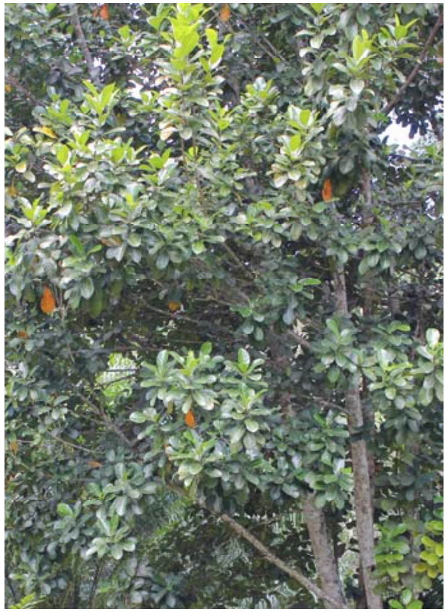

107

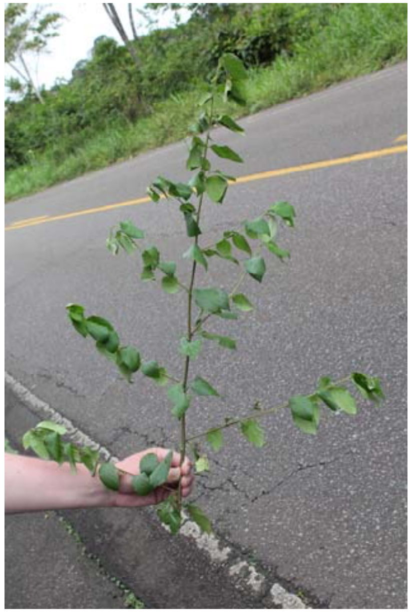

125

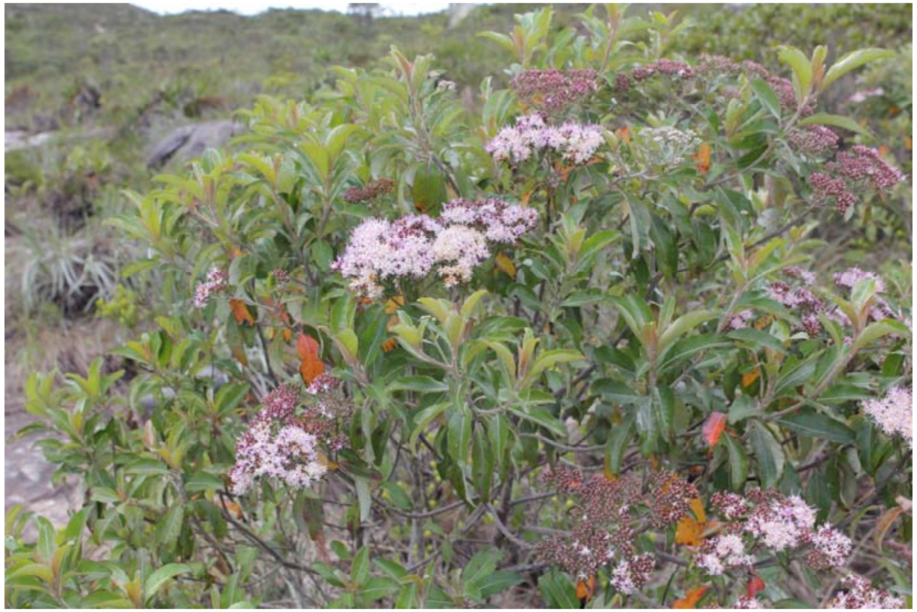

130

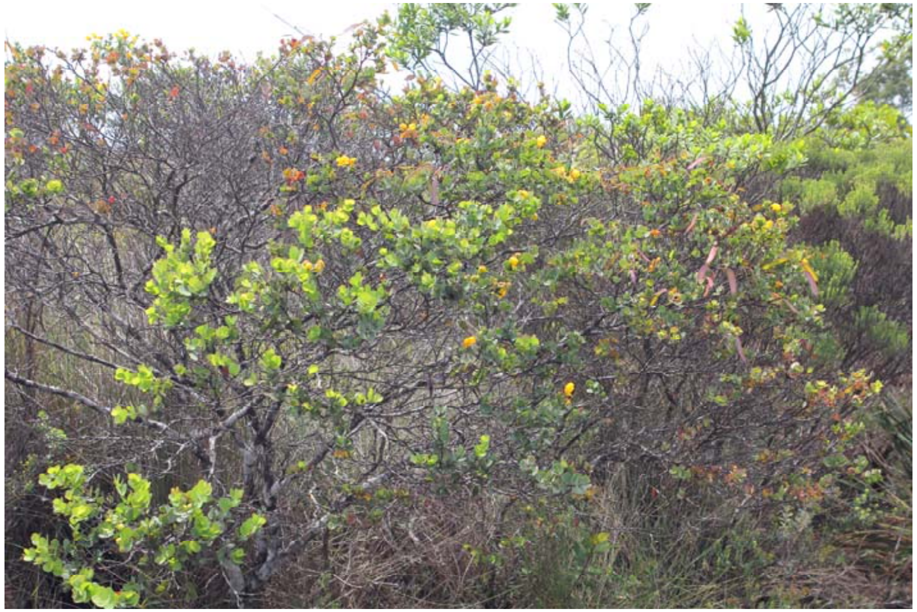

163

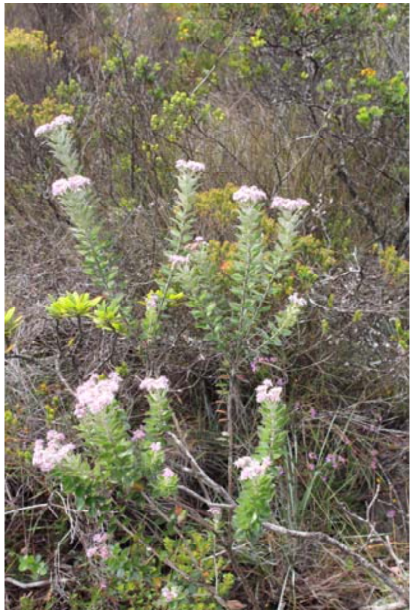

164

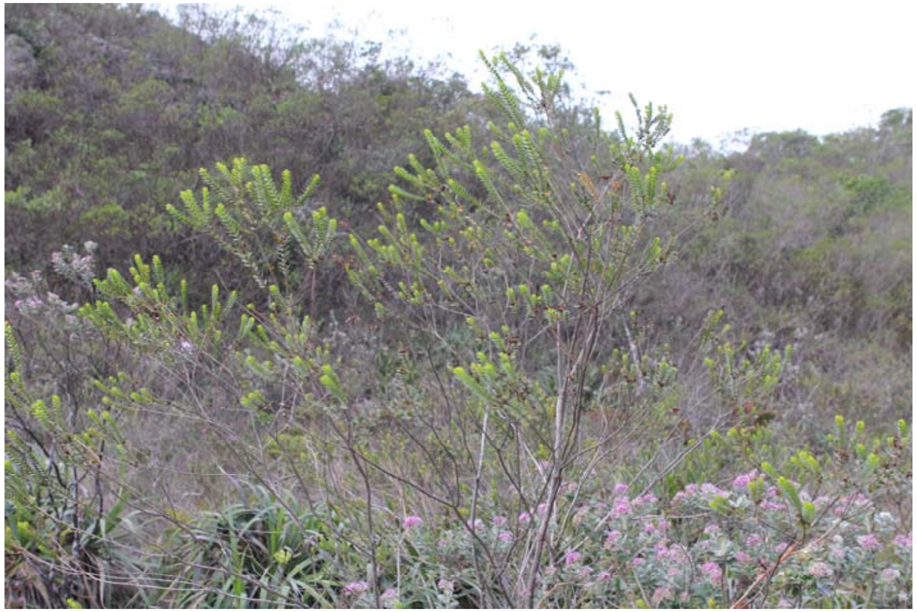

166

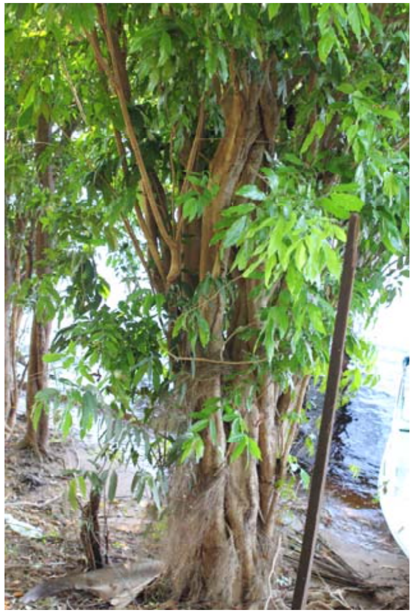

283

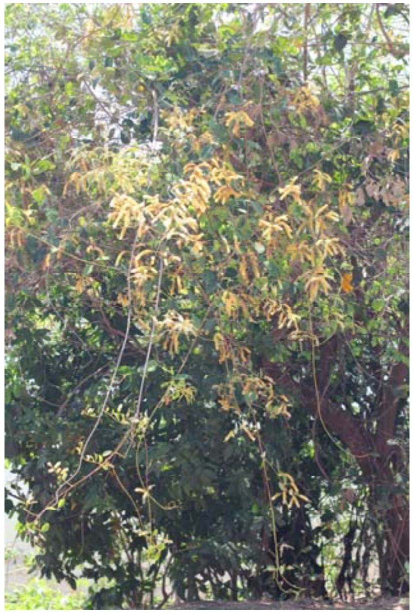

471

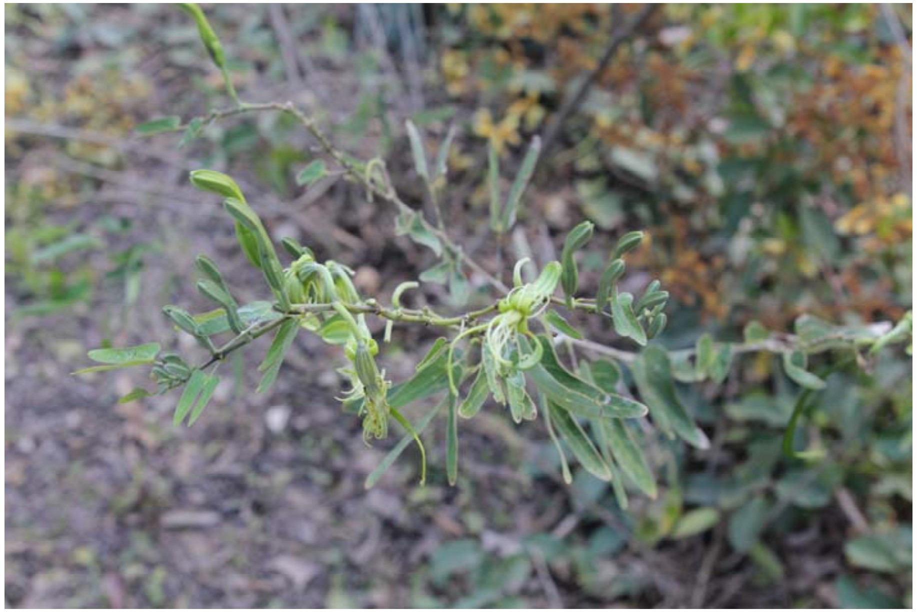

473
