# Supplementary material for: Confirmation bias leads to overestimation of losses of woody plant foliage to insect herbivores in tropical regions
Source: PeerJ. 2014 Dec 23;2:e709. doi: 10.7717/peerj.709 (PMC4277485; doi:10.7717/peerj.709)
Supplement: Appendix S3 [file peerj-02-709-s003.pdf]

Appendix S3. Foliar damage of woody plants measured from nature, wildlife and portrait photographs[1].

Photo #	Locality	Habitat	Plant species[2]	Foliar damage[3]		
				n	dam	cons
072	Diamantina	Subalpine shrubland		93	6.8	0.17
075	Diamantina	Subalpine shrubland		54	28.3	2.74
076	Diamantina	Subalpine shrubland	Clusiaceae, *Clusia* sp.	147	11.4	0.49
089	Diamantina	Subalpine shrubland		39	18.0	0.25
091	Diamantina	Subalpine shrubland		154	15.0	1.68
100	Diamantina	Subalpine shrubland	Melastomataceae, *Henriettella* sp.	137	6.4	0.78
130	Diamantina	Subalpine shrubland	Melastomataceae	114	11.2	0.61
176	Novo Airão	Riverside		8	0	0
198	Novo Airão	Riverside		38	26.9	3.60
218	Novo Airão	Forest		18	4.6	0.06
219	Novo Airão	Riverside		33	13.4	0.34
225	Novo Airão	Forest (submerged)		9	7.9	3.99
4255	Ilhéus	Atlantic forest	Melastomataceae, *Henriettella* sp.	38	52.9	5.34
4300	Ilhéus	Atlantic forest		14	63.5	2.66
4326	Ilhéus	Atlantic forest	Malvaceae, *Theobroma cacao*	135	38.2	2.22
4343	Diamantina	Subalpine shrubland	Clusiaceae, *Clusia* sp.	80	5.5	0.65
4351	Diamantina	Subalpine shrubland		92	5.0	0.14
4378	Diamantina	Subalpine shrubland	Melastomataceae	70	3.8	0.20
4407	Diamantina	Forest		28	4.7	0.82
4407	Diamantina	Forest		61	12.2	0.77
4439	Diamantina	Forest		102	5.6	0.42
4471	Novo Airão	Forest (at a roadside)		67	16.2	0.82
4541	Novo Airão	Forest (submerged)		55	2.4	0.04
4596	Novo Airão	Riverside	Myrtaceae, *Syzygium* sp.	92	3.3	0.05
4726	Novo Airão	Riverside	Rubiaceae, *Warszewiczia* sp.	23	36.6	1.91
4777	Novo Airão	Forest (at a roadside)	Euphorbiaceae	44	76.9	1.84
5039	Pantanal	Roadside		90	72.6	10.62
5226	Pantanal	Roadside		81	14.0	0.86
5237	Pantanal	Roadside		77	6.6	0.32
5342	Pantanal	Roadside		53	13.7	0.60

[1] High-resolution images available from authors upon request.
[2] Identified from a photograph by K. Ruokalainen.
[3] Mean values based on three independent measurements (see text for details). n, number of leaves; dam, proportion of leaves damaged by defoliating insects (%); cons, proportion of leaf area consumed by defoliating insects (%).

Photographs

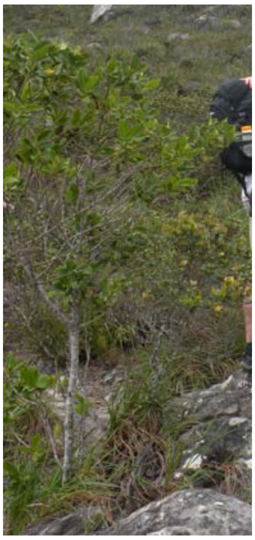

72

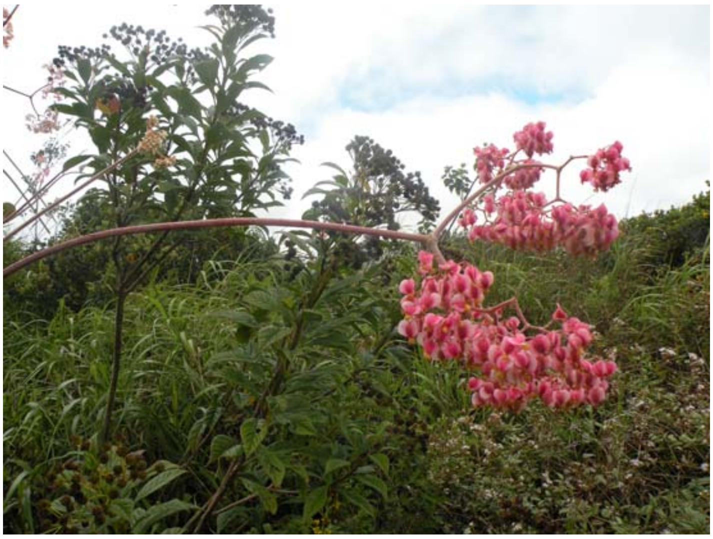

075

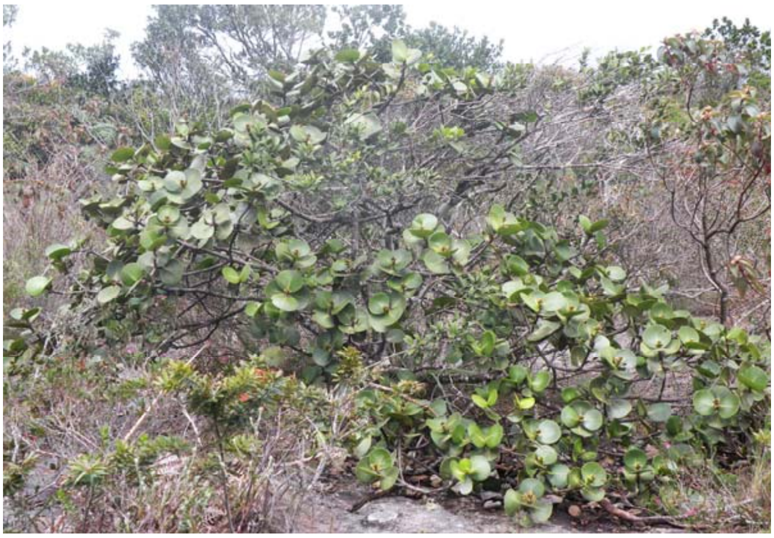

076

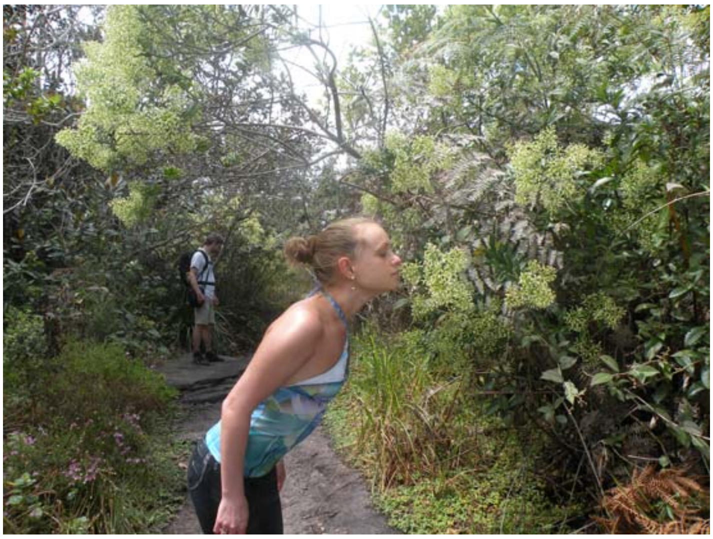

089

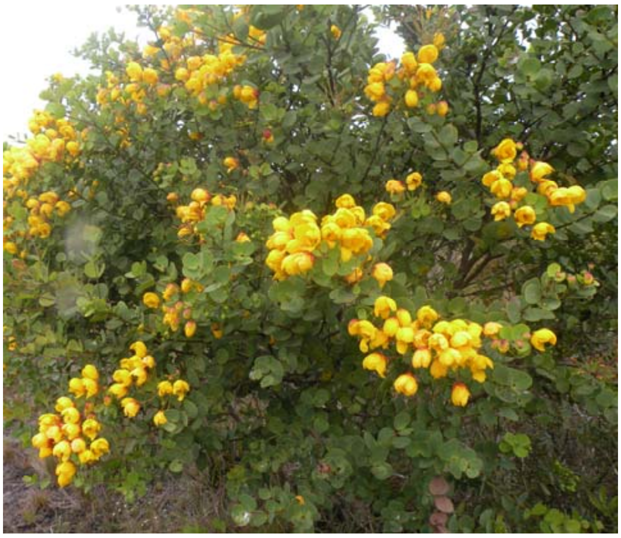

091

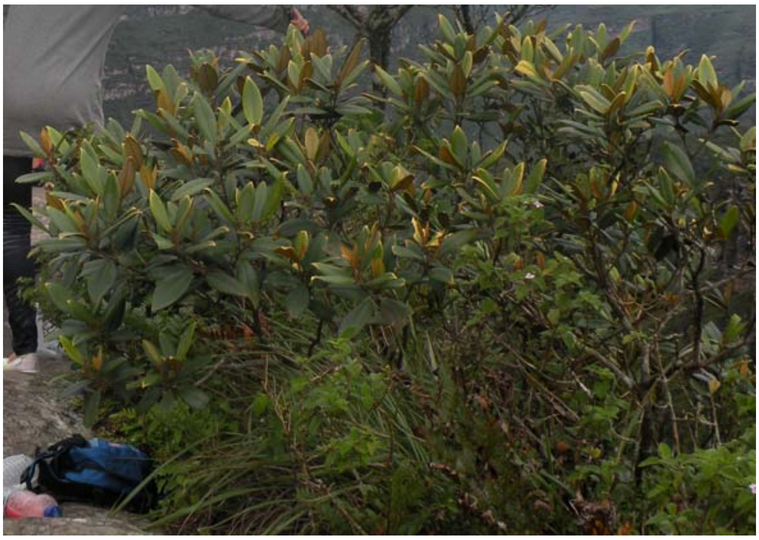

100

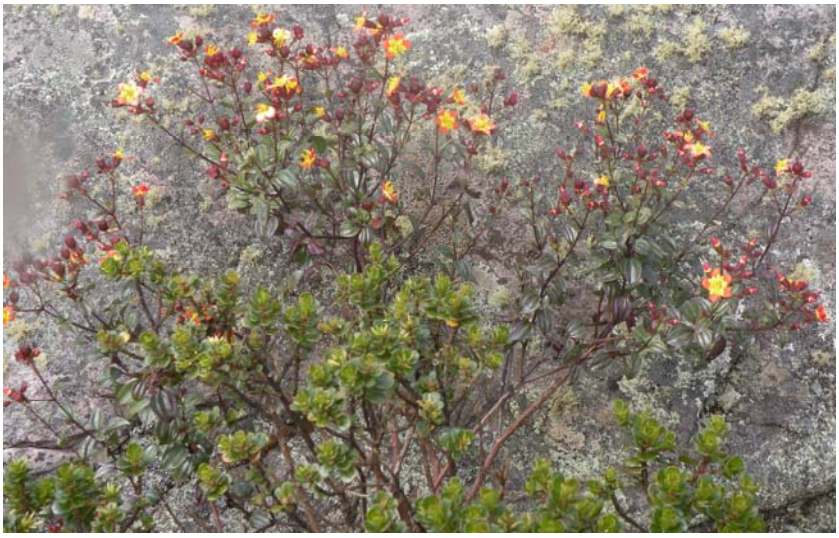

130

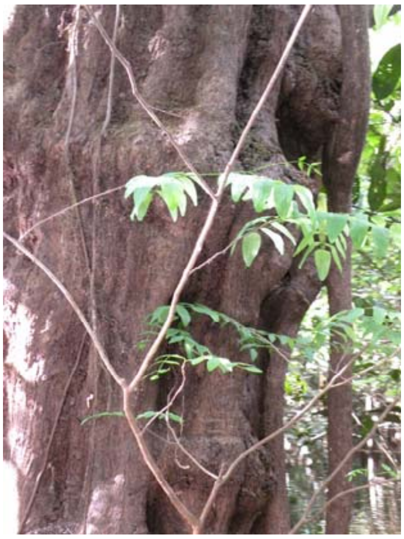

176

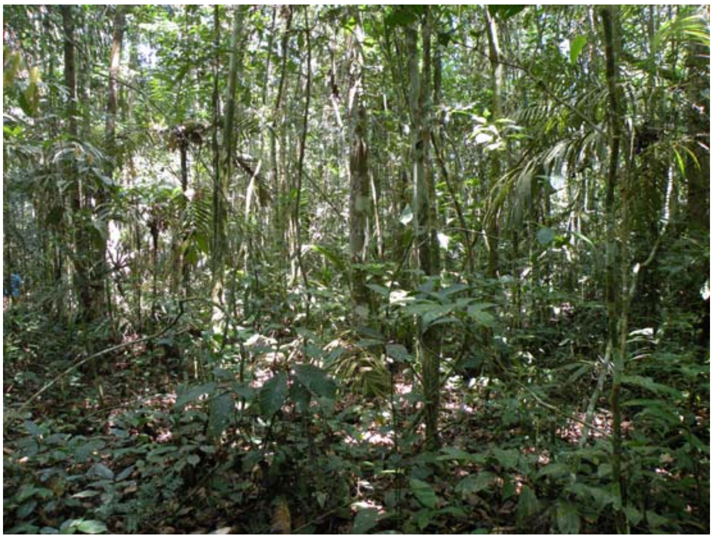
198

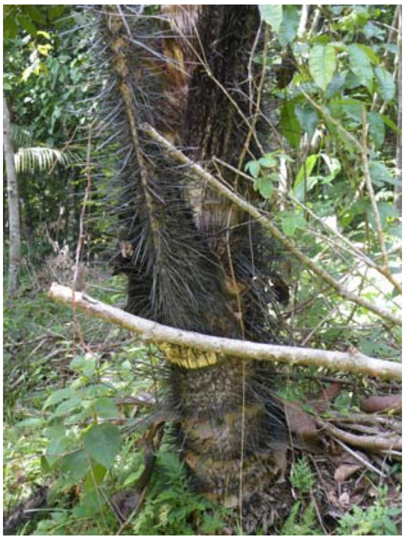
218

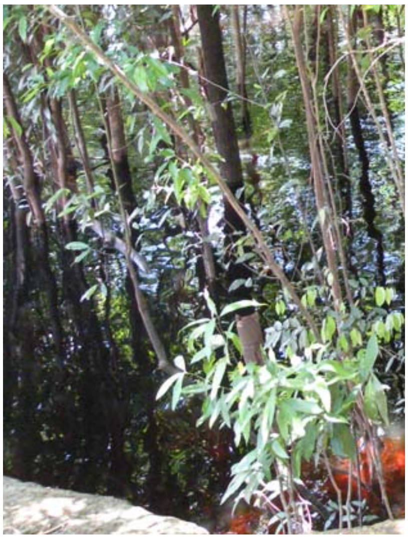
219

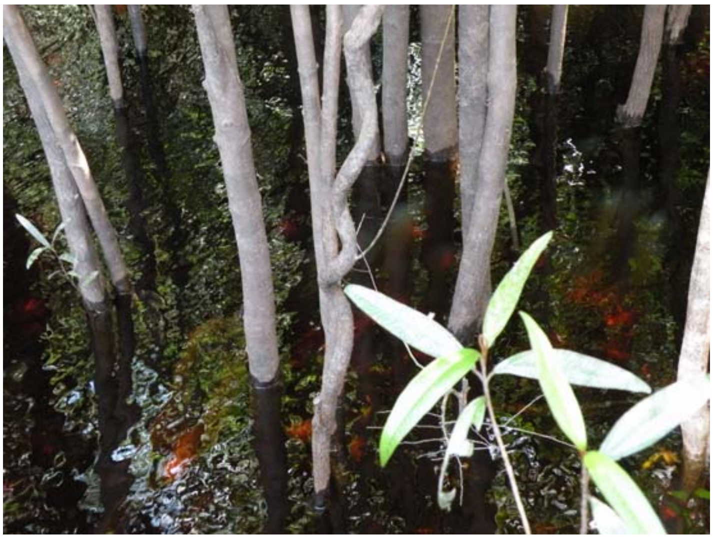
225

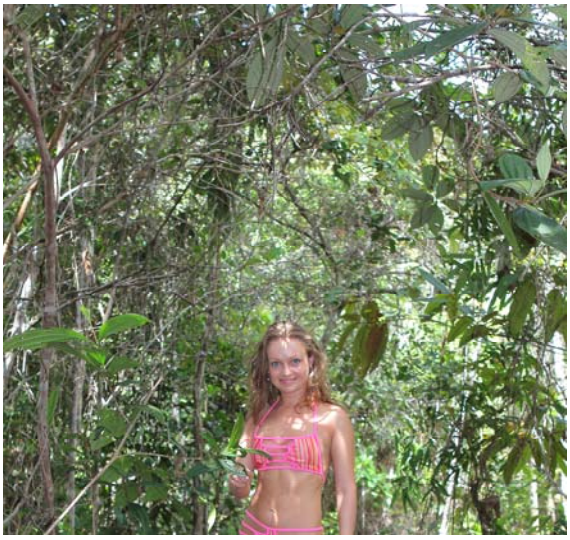
4255

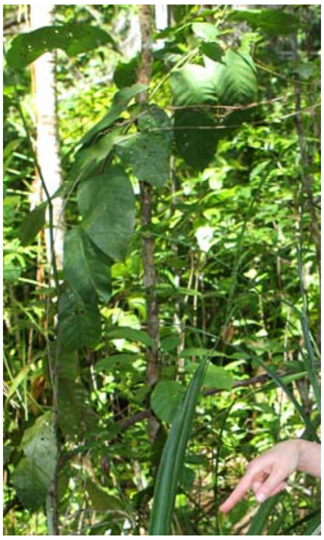
4300

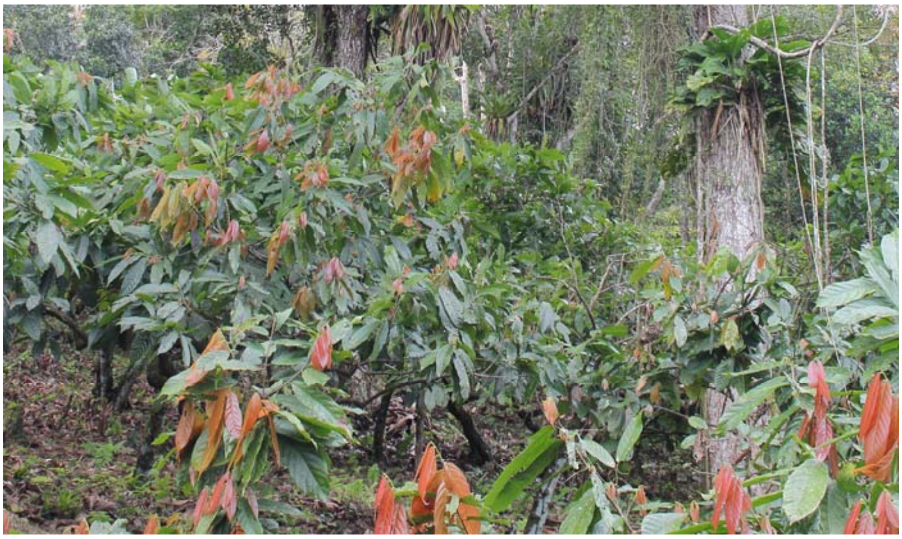

4326

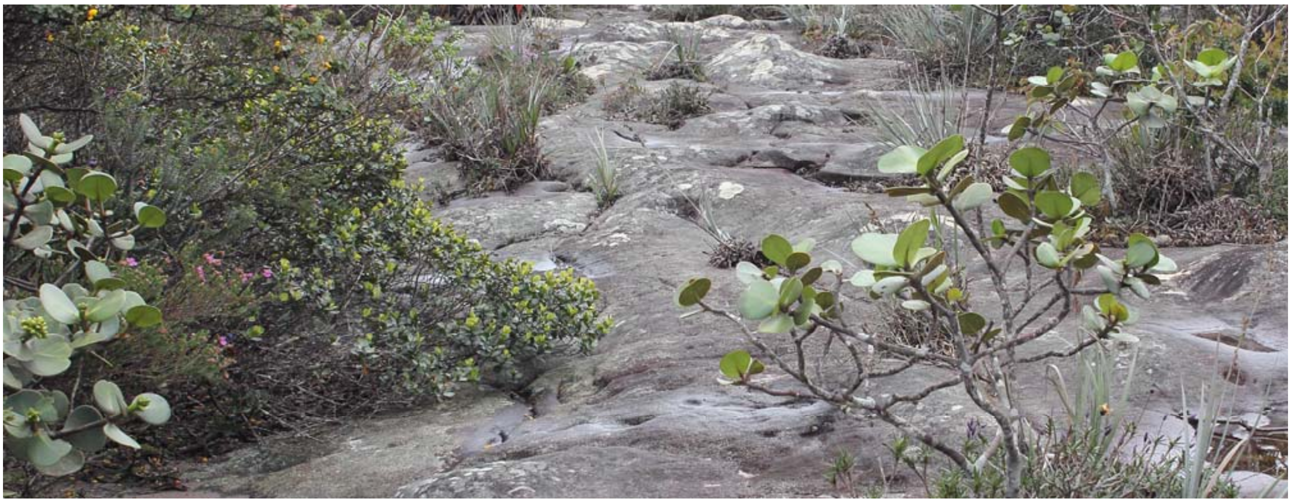

4343

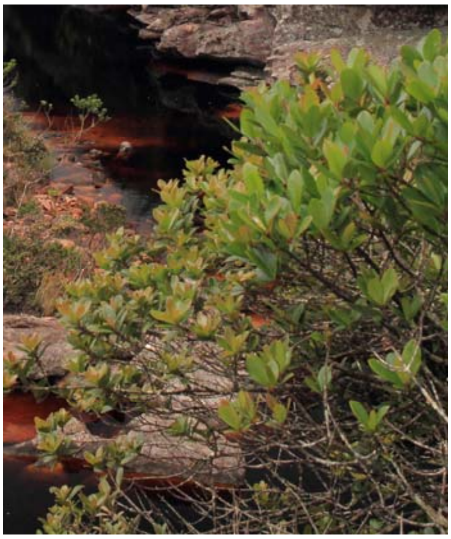

4351

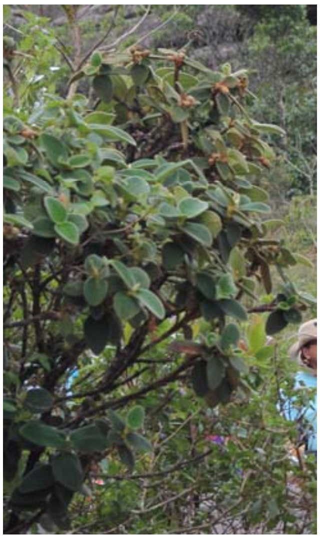

4378

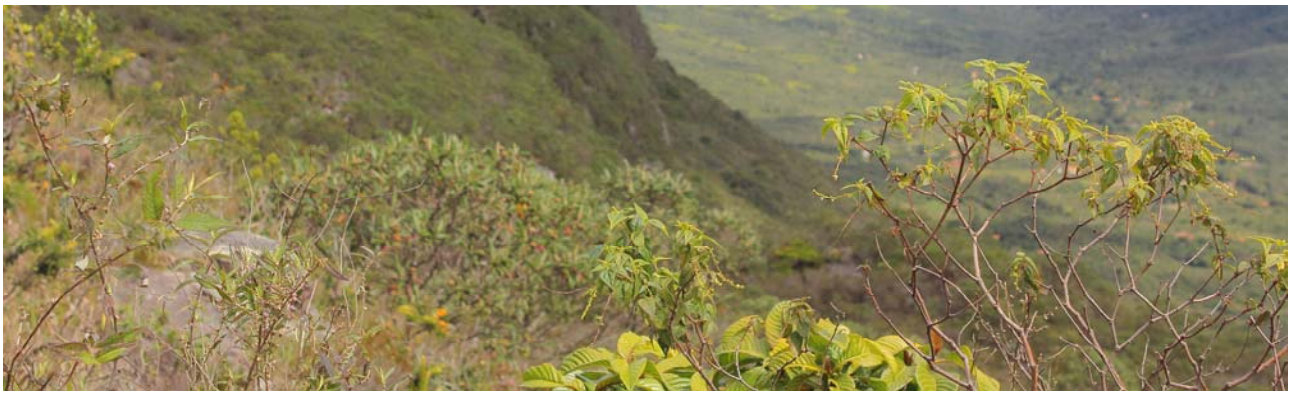
4407

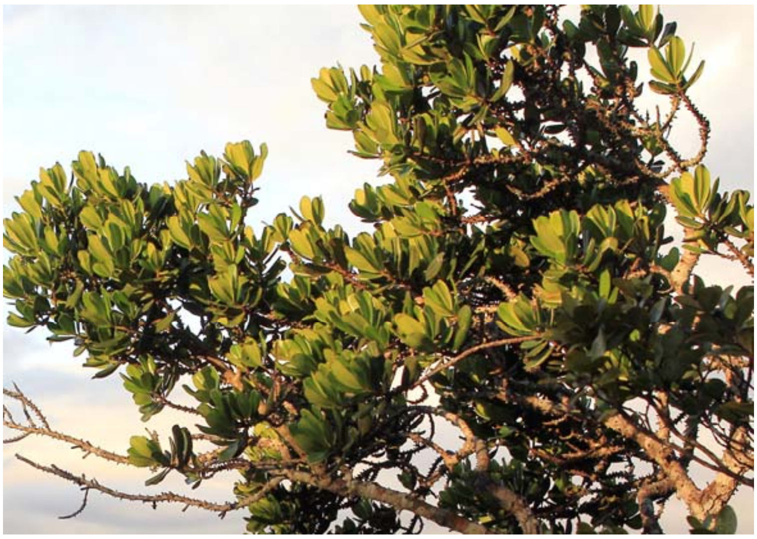
4439

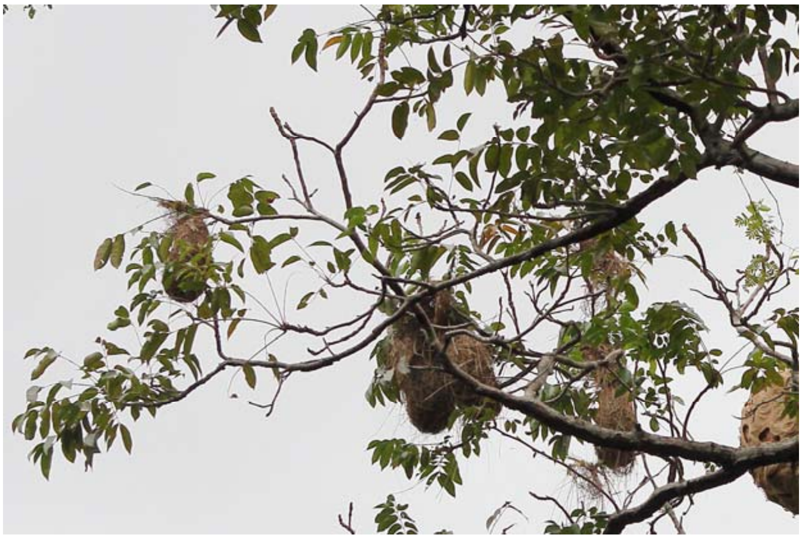
4471

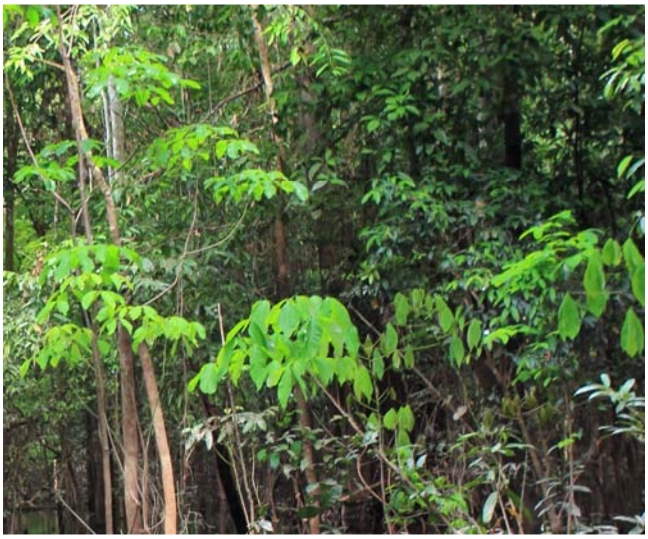

4541

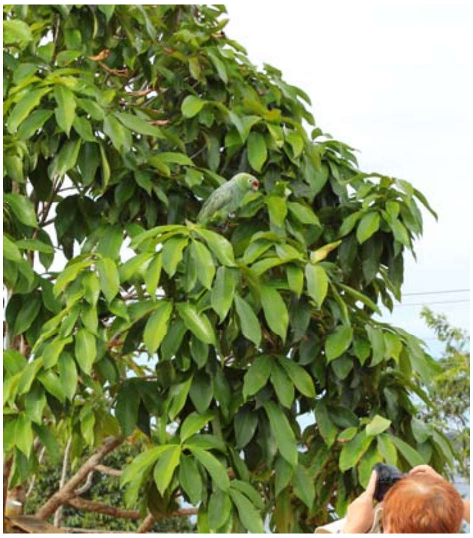

4596

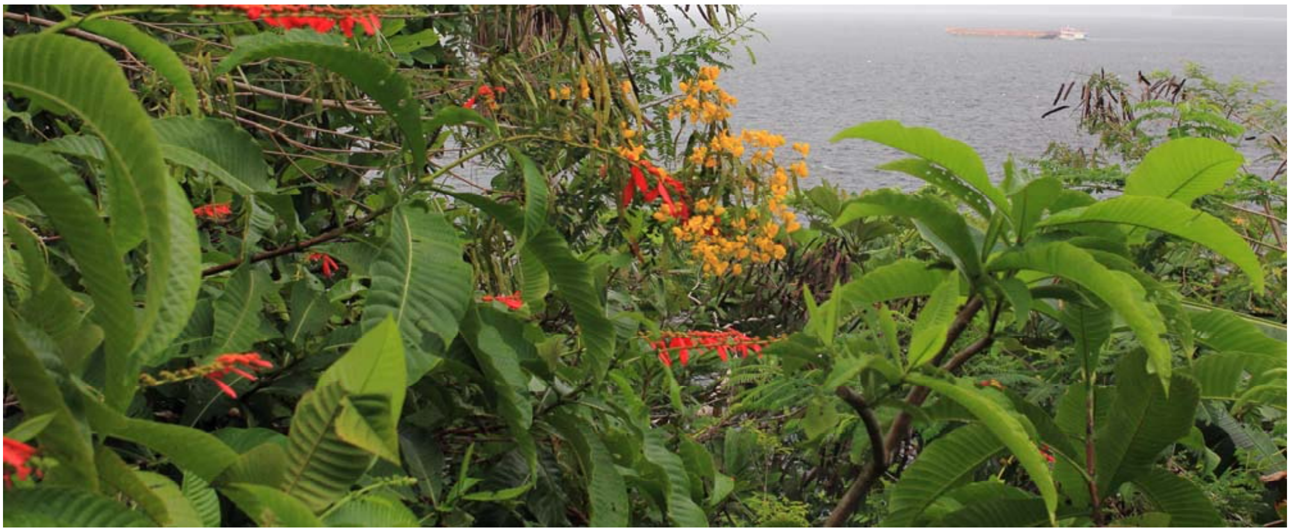

4726

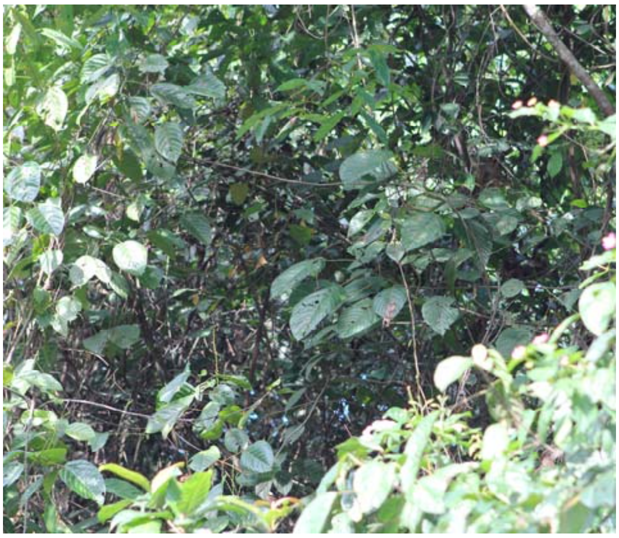

4777

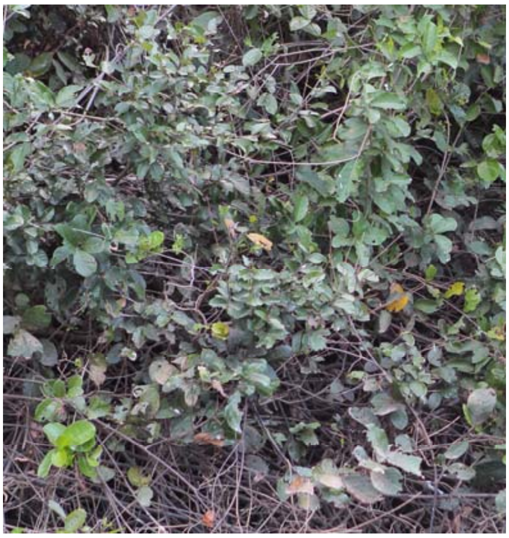

5039

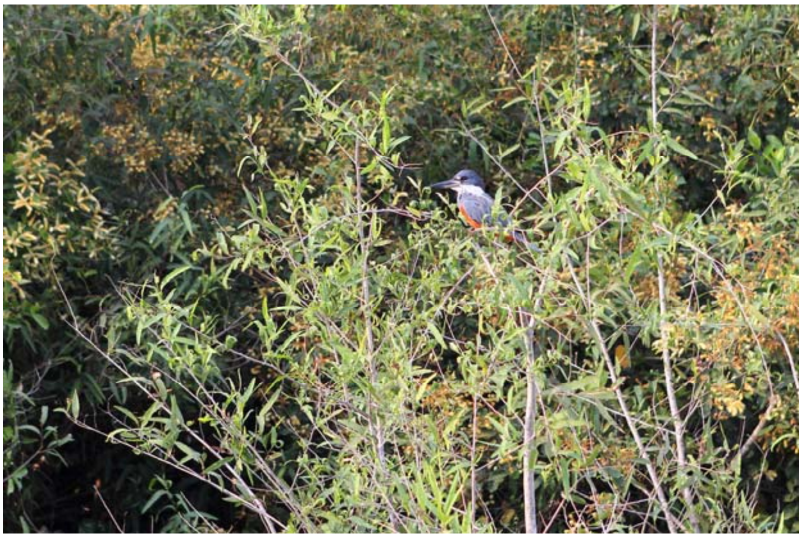

5226

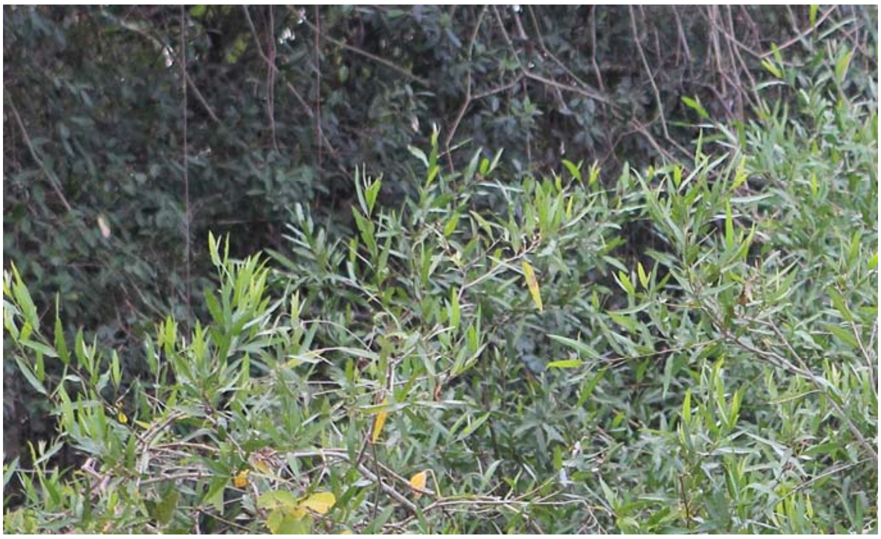

5237

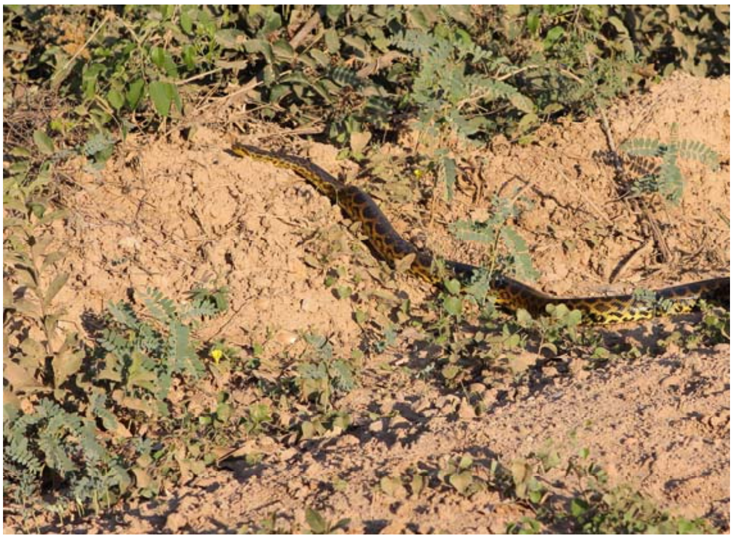

5342
