# Supplementary material for: Confirmation bias leads to overestimation of losses of woody plant foliage to insect herbivores in tropical regions
Source: PeerJ. 2014 Dec 23;2:e709. doi: 10.7717/peerj.709 (PMC4277485; doi:10.7717/peerj.709)
Supplement: Appendix S4 [file peerj-02-709-s004.pdf]

Appendix S4. Foliar damage of woody plants measured from nature and wildlife photographs found in the WWW.

**WWW page**	**Locality**	**Foliar damage**[1]		
		n	dam	cons
http://eco-turizm.net/wp-content/uploads/2012/09/водная-пустыня-Бразилия.jpg	Lencois Maranhenses	19	15.8	0.21
http://www.1zoom.ru/Природа/обои /337825/z2333.3/	Iguassu Falls	51	41.2	1.74
http://www.1zoom.ru/Природа/обои/373787/z3655.9/	Iguassu Falls	25	24.0	1.00
http://www.1zoom.ru/Природа/обои/337970/z2655.7/	Iguassu Falls	29	17.2	0.67
http://img1.liveinternet.ru/images/attach/c/6/91/785/91785683_large_supercoolpics_02_19092012221630.jpg	NE Brazil	15	13.3	1.20
http://www.brasileiro.ru/cerrado/images/IMG_0648_JPG.jpg	Goiás	26	46.2	4.23
http://www.brasileiro.ru/cerrado/images/IMG_0437_JPG.jpg	Goiás	36	16.7	0.56
http://www.brasileiro.ru/cerrado/images/IMG_0545_JPG.jpg	Goiás	67	6.0	0.28
http://www.brasileiro.ru/cerrado/images/IMG_0566_JPG.jpg	Goiás	14	14.3	1.11
http://www.brasileiro.ru/cerrado/images/IMG_0577_JPG.jpg	Goiás	35	11.4	0.13
http://blog.kupibilet.ru/strashnie-tainy-planeta-zemlia/	Ilha da Queimada Grande	14	21.4	0.11
http://www.brasileiro.ru/piri2/pages/CIMG1477_JPG.htm	Near Pirenópolis, Goiás	45	35.6	2.50
http://www.brasileiro.ru/piri2/pages/CIMG1482_JPG.htm	Near Pirenópolis, Goiás	29	13.8	1.93
http://www.brasileiro.ru/piri2/pages/CIMG1495_JPG.htm	Near Pirenópolis, Goiás	28	3.6	0.11
http://www.brasileiro.ru/chapada/pages/Chapada-dos-Veadeiros-20020_jpg	Chapada dos Veadeiros	19	15.8	0.97
http://www.brasileiro.ru/chapada/pages/Chapada-dos-Veadeiros-20046_jpg.htm	Chapada dos Veadeiros	18	0	0
http://www.brasileiro.ru/pirenopolis/pages/IMG_1253_JPG.htm	Near Pirenópolis, Goiás	13	0	0
http://www.brasileiro.ru/pirenopolis/pages/IMG_1321_JPG.htm	Near Pirenópolis, Goiás	21	42.9	1.02
http://www.brasileiro.ru/pir/pages/Picture 20011_jpg.htm	Near Pirenópolis, Goiás	12	50.0	1.67
http://forum.awd.ru/gallery/images/upload/675/71f/67571f1b1a34412fca9111aced1a747a.jpg	Lençóis Maranhenses National Park	12	16.7	0.08
http://sabibon.info/6063-lesa-amazonki-foto.html (Amazonas25)	Unknown (Amazon basin)	17	11.8	0.35
http://sabibon.info/6063-lesa-amazonki-foto.html (Amazonas15)	Unknown (Amazon basin)	17	11.8	0.20
http://zastavok.ru/priroda/leto/308-lesa_brazilii.html	Unknown	17	0	0
http://images.sciencedaily.com/2014/08/140828142656-large.jpg	Unknown (Atlantic coast)	21	19.0	0.90
http://jwblackboard.com/amazon-rainforest-animals-and-plants-facts-218.jpg	Unknown (Amazon basin)	18	0	0

[1] Mean values based on three independent measurements (see text for details). n, number of leaves; dam, proportion of leaves damaged by defoliating insects (%); cons, proportion of leaf area consumed by defoliating insects (%).

http://2.bp.blogspot.com/-dUK5UyW34Lo/Uwe_zRMMv9I/AAAAAAAAAGc/ NOlmDI95g1M/ s1600/Amazon+Rainforest+Animals+-+Spider+Monkey.jpg	Unknown (Amazon basin)	28	0	0
http://www.fotothing.com/LuisBacher/photo/9a269957eaf01d56596979d1f09d6c71/	Unknown[2]	31	12.9	0.15
https://sunergoscoffee.com/wordpress/wp-content/uploads/wpsc/product_images/Brazil-tree-in-flower.JPG	Unknown	49	8.2	0.09
http://www.mirkrasiv.ru/nature/zhabotikaba-ili-dzhabotikaba-lat-myrciaria-caulifloria-brazilskoe-vinogradnoe-derevo.html	Unknown[3]	16	0	0
http://photo.qip.ru/users/demavi/150912930/166620723/	Unknown (Amazon basin)	26	42.3	0.69

[2] Plant name: *Lophantera lactescens*.
[3] Plant name: *Myricaria cauliflora*.
